# Combination of a New Oral Demethylating Agent, OR2100, and Venetoclax for Treatment of Acute Myeloid Leukemia

**DOI:** 10.1158/2767-9764.CRC-22-0259

**Published:** 2023-02-21

**Authors:** Kazuharu Kamachi, Hiroshi Ureshino, Tatsuro Watanabe, Nao Yoshida-Sakai, Yuki Fukuda-Kurahashi, Kazunori Kawasoe, Toshimi Hoshiko, Yuta Yamamoto, Yuki Kurahashi, Shinya Kimura

**Affiliations:** 1 Department of Drug Discovery and Biomedical Sciences, Faculty of Medicine, Saga University, Saga, Japan.; 2 Division of Hematology, Respiratory Medicine and Oncology, Department of Internal Medicine, Faculty of Medicine, Saga University, Saga, Japan.; 3 Department of Hematology and Oncology, Research Institute for Radiation Biology and Medicine, Hiroshima University, Hiroshima, Japan.; 4 OHARA Pharmaceutical Co., Ltd, Tokyo, Japan.

## Abstract

The standard treatment for elderly patients with acute myeloid leukemia (AML) is venetoclax (Ven), a BCL-2–selective inhibitor, combined with hypomethylating agents (HMA) such as azacitidine or decitabine. This regimen results in low toxicity, high response rates, and potentially durable remission; however, because of low oral bioavailability, these conventional HMAs must be administered intravenously or subcutaneously. A combination of oral HMAs and Ven would provide a therapeutic advantage over parenteral administration of drugs and improve quality of life by reducing the number of hospital visits. Previously, we showed the promising oral bioavailability and antileukemia effects of a new HMA, OR2100 (OR21). Here, we investigated the efficacy and underlying mechanism of OR21 when used in combination with Ven to treat AML. OR21/Ven showed synergistic antileukemia effects *in vitro,* and significantly prolonged survival without increasing toxicity in a human leukemia xenograft mice model. RNA sequencing following combination therapy revealed downregulation of *VAMP7*, which is involved in autophagic maintenance of mitochondrial homeostasis. Combination therapy led to accumulation of reactive oxygen species, leading to increased apoptosis. The data suggest that the combination of OR21 plus Ven is a promising candidate oral therapy for AML.

**Significance:**

The standard treatment for elderly patients with AML is Ven combined with HMAs. OR21, a new oral HMA plus Ven showed synergistic antileukemia effects *in vitro* and *vivo*, suggesting that the combination of OR2100 plus Ven is a promising candidate oral therapy for AML.

## Introduction

Acute myeloid leukemia (AML) is a clonal hematopoietic stem cell and progenitor cell disorder caused by acquired and/or occasionally inherited genetic and/or epigenetic alterations (1). Although 80% of patients with AML achieve complete remission after receiving conventional chemotherapy (2), the majority eventually relapse due to clonal evolution, aberrant DNA methylation, and persistence of leukemic stem cells following chemotherapy.

The median age at diagnosis is 68 years (3). Because these patients with AML generally have several comorbidities, they are often ineligible for intensive chemotherapy or allogenic stem cell transplantation, meaning that they receive less intensive chemotherapy regimens such as hypomethylating agents (HMA) or low-dose cytarabine (LDAC; ref. 4). Combining Venetoclax (Ven), a selective BCL-2 inhibitor, with HMAs or LDAC yields high response rates and durable remission, with a good safety profile, in patients with AML who are ineligible for intensive chemotherapy (3–5). Thus, treatment with Ven plus HMAs or LDAC is the standard treatment strategy for patients with AML (6).

HMAs such as azacitidine (AZA) or decitabine (DAC) must be administered parenterally because they are easily degraded by cytidine deaminase (CDA). Orally bioavailable HMAs would provide a therapeutic advantage over HMAs that have to be administered via the parenteral route by improving quality of life through a reduction in the number of hospital visits (7). Currently, two orally bioavailable HMAs, CC-486 (oral azacitidine) and ASTX727 (decitabine + cedazuridine), were approved by the FDA in 2020 (8, 9).

Previously, we developed a new HMA, OR2100 (OR21), which resists degradation by CDA, and demonstrated its favorable oral bioavailability and antileukemia effects, along with low toxicity (10–12). Furthermore, OR21 is a single compound, giving it enormous advantages with respect to ease of production and quality control. Here, we evaluated the preclinical efficacy of OR21 plus Ven as a treatment for AML.

## Materials and Methods

### Reagents

OR21 was synthesized by OHARA Pharmaceutical Co. AZA and DAC were purchased from Sigma-Aldrich. Ven and P62-mediated mitophagy inducer (PMI) were purchased from MedChemExpress. S63845, a selective myeloid cell leukemia-1 (MCL-1) inhibitor, and Z-VAD-FMK (Z-VAD) were purchased from Selleck Chemicals. Rapamycin (Rapa) was purchased from Tokyo Chemical Industry. All reagents were dissolved in DMSO and stored at −20°C.

### Cell Lines and Culture

SKM1 cells (derived from the blast cells of a patient with myelodysplastic syndrome) were purchased from the Japanese Collection of Research Bioresources Cell Bank. HL60, THP-1, KG1a, and Kasumi-1 cell lines (derived from patients with AML) were purchased from the ATCC. SKM1, THP-1, and Kasumi-1 cells were maintained in RPMI1640 medium (Sigma-Aldrich) containing 10% FBS (Sigma-Aldrich). HL60 and KG1a cells were maintained in Iscove's Modified Dulbecco's Medium (Sigma-Aldrich) supplemented with 20% FBS. All cell lines were used within 2 months after thawing or within 25 passages, and were authenticated by short tandem repeat DNA profiling analyses. All cultures were checked regularly for *Mycoplasma* infection.

### Cell Growth Assay and Determination of Synergistic Drug Effects

Cell proliferation was evaluated in a CCK-8 assay (Dojindo), and IC_50_ were determined by nonlinear regression using CalcuSyn software (Biosoft). All procedures for cell proliferation analyses were conducted in accordance with the manufacturers’ instructions. The synergistic Bliss scores were determined using the freely available online SynergyFinder software (13).

### Apoptosis Assays

Cells were incubated for 72 hours with various concentrations of each compound, and then stained with APC-conjugated Annexin V (BioLegend) and propidium iodide (Sigma-Aldrich). Apoptotic cells, defined as APC-conjugated Annexin V-positive cells, were analyzed using a FACSVerse cytometer (BD Biosciences). Data are expressed as the mean ± SD of three independent experiments and were analyzed using FlowJo software (Tree Star).

### Western Blot Analysis

Whole-cell lysates were extracted from cell lines treated with the indicated compounds using RIPA buffer containing phenylmethylsulfonylfluoride, a protease inhibitor, sodium orthovanadate (Santa Cruz Biotechnology), and phosphatase inhibitors (Sigma-Aldrich). Protein concentrations were determined using the Bio-Rad protein assay (Bio-Rad). Equal amounts of whole-cell lysate were denatured (5 minutes, 95°C) in NuPAGE LDS sample buffer (Invitrogen) and separated on NuPAGE 3%–8% Tris-acetate gels or 4%–12% Bis-Tris gels (Invitrogen) prior to transfer to nitrocellulose membranes (LI-COR Biotechnology). Immunoblotted bands were detected using the ECL detection reagent (GE Healthcare). The following primary antibodies were used: rabbit polyclonal anti-BCL-2, anti-MCL-1, anti-BCL-xL, anti-BIM, anti-VAMP7, anti-RNH1, anti-p62 (Proteintech), anti-tubulin (Cell Signaling Technology), and anti-LC3B (Abcam).

### RNA Sequencing

Total RNA was isolated from HL60 and KG1a cells treated with vehicle (DMSO; cont), or with 1.0 μmol/L OR21, or 0.1 μmol/L (HL60) or 0.5 μmol/L (KG1a) Ven, or OR21 (1.0 μmol/L) plus Ven (0.1 or 0.5 μmol/L) using the Direct-zol RNA MiniPrep kit (Zymo Research). The amount and quality of RNA were evaluated using a NanoDrop ND-2000 (Thermo Fisher Scientific). RNA extraction, mRNA isolation, library preparation, sequencing using the NovaSeq 6000 system (Illumina), and analysis of differentially expressed genes (DEG) was performed by Rhelixa Inc.

### Public Dataset Analyses

RNA-sequencing data from patients with AML who were treated with Ven plus HMAs or Ven plus cytarabine were analyzed alongside the patient's clinical response data. The data were obtained from the European Genome-phenome Achieve (EGA; EGAS00001003820; ref. 14).

### Measurement of the Mitochondrial Membrane Potential

The mitochondrial membrane potential (MMP) was measured using the MT-1 MitoMP Detection Kit (Dojindo). Briefly, HL60 and KG1a cells were stained with MT-1 Dye (30 minutes, 37°C) and then incubated for 6 hours with vehicle, or with 1.0 μmol/L OR21, 0.1 μmol/L (HL60), or 0.5 μmol/L (KG1a) Ven, and OR21 plus Ven. MMP was analyzed using a FACSVerse cytometer (BD Biosciences). Data are presented as the mean ± SD of three independent experiments and were analyzed using FlowJo software.

### Lentiviral Preparation and Infection

Lentiviral particles used for the transduction of sh-VAMP7 cDNA [VB220803-1883vcw, pLV (shRNA)-EGFPU6>hVAMP7] and control lentiviral particles [VB010000-0009mxc, pLV (shRNA)-EGFP:T2A:Puro-U6>Scramble_shRNA] were prepared by VectorBuilder. The HL60 and KG1a cell lines were infected with lentiviral particles on untreated plates coated with RetroNectin (Takara Bio).

### Measurement of Reactive Oxygen Species

Reactive oxygen species (ROS) and mitochondrial ROS (mtROS) levels were measured using the CellROX Green Flow Cytometry Assay Kit (Thermo Fisher Scientific) and mtSOX Deep Red (Dojindo), respectively. Briefly, AML cell lines were cultured in 24-well plates at a density of 1 × 10^6^ cells/well and then incubated for 12 or 48 hours with various concentrations of vehicle (DMSO), or with 1.0 μmol/L OR21, 0.1 μmol/L (HL60) or 0.5 μmol/L (KG1a) Ven, or OR21 plus Ven, after which ROS were detected by flow cytometry. Mean fluorescence intensity (MFI) was analyzed by FlowJo software.

### Detection of Mitophagy

The Mitophagy Detection Kit (Dojindo) was used to detect mitophagy. Briefly, HL60 and KG1a (5 × 10^5^ cells) were preincubated with Mitophagy Dye (30 minutes, 37°C), and then washed with Hank's Balanced Salt Solution (HBSS). The cells were then incubated for 6 hours with medium containing vehicle (DMSO), 5.0 μmol/L (HL60) or 10 μmol/L (KG1a) OR21, or 0.5 μmol/L (HL60) or 5.0 μmol/L (KG1a) Ven, or OR21 plus Ven (5.0 μmol/L+ 0.5 μmol/L or 10 μmol/L + 5.0 μmol/L). Next, 1 μmol/L of Lyso Dye was added for 30 minutes. After washing with HBSS, mitophagy was detected by flow cytometry or observed under a confocal microscope (LSM880; ZEISS). MFI was analyzed using FlowJo software.

### Xenograft Mouse Models

NOG (NOD/Shi-scid/IL-2Rγ^null^) mice were obtained from In-Vivo Science Inc.. Six-week-old female NOG mice received an intravenous injection of 5 × 10^6^ HL60 cells via the tail vein. Mice were then randomized into six groups (vehicle; 1% DMSO and dissolution of VEN; OR21, 2.7 mg/kg; OR21, 5.4 mg/kg; Ven, 25 mg/kg; OR21, 2.7 mg/kg plus Ven 25 mg/kg; or OR21, 5.4 mg/kg plus Ven, 25 mg/kg). OR21 was dissolved in DMSO and diluted with 10% cyclodextrin. Ven was formulated in phosal 50 mixture solution comprising 60% phosal 50 propylene glycol (H. Holstein), 30% polyethylene glycol 400, and 10% ethanol. Mice received vehicle (DMSO, injected intraperitoneally twice a week; 100 μL of phosal 50 mixture solution, oral gavage, once per day), OR21 (intraperitoneally, twice a week), and Ven (oral gavage, once daily) from day 7 to day 28 postinjection of HL60 cells. At day 28, peripheral blood samples from each mouse were obtained and the percentage of human CD45^+^ cells was analyzed by flow cytometry.

KG1a cells (1 × 10^7^ cells/mouse) were subcutaneously inoculated at the dorsal site into 6-week-old female NOG mice. The mice were then randomized into four groups (vehicle; 1% DMSO and dissolution of VEN; OR21 5.4 mg/kg, intraperitoneally, twice a week; Ven 50 mg/kg, oral gavage, once daily; OR21 5.4 mg/kg plus Ven 50 mg/kg, *n* = 6 in each group). Treatment was initiated from day 10 after inoculation and lasted 10 days. Tumor volumes, calculated as (short axis)^2^ × (long axis)/2, were measured twice a week. All mice were euthanized on day 21 after inoculation and the xenotransplanted tumors isolated from each mouse were weighted.

The intraperitoneal route was used because OR21 is easily degraded by gastric acid. Gelatin capsules filled with enteric-coated granules of OR21 were prepared and oral availability was confirmed in cynomolgus monkeys, as described previously (11); however, the granules were too large to administer to mice. Mice were observed daily and euthanized when they showed signs of progressive disease, including hind limb paralysis, >20% weight loss, or lethargy. All animal experiments were approved by the Institutional Review Board of Saga University and performed according to the university's institutional guidelines.

### Statistical Analysis

All data are expressed as the mean ± SD, and significant differences between two groups were determined using Student *t* test and the Mann–Whitney *U* test. Survival curves were estimated using the Kaplan–Meier method and compared using the log-rank test. Pearson correlation analysis was used to assess the linear relationship between two variables. The Kruskal–Wallis test and one-way ANOVA were used when comparing four groups, and Bonferroni correction was applied when comparing two groups. *P* < 0.05 was considered statistically significant. All statistical analyses were performed using the EZR software package (Saitama Medical Center, Jichi Medical University, Saitama, Japan).

### Data Availability

RNA-sequencing data are available from the DNA Data Bank of Japan under accession number DRA015481.

## Results

### Combination of HMAs plus Ven has Strong Antileukemia Effects Against AML

**FIGURE 1:**
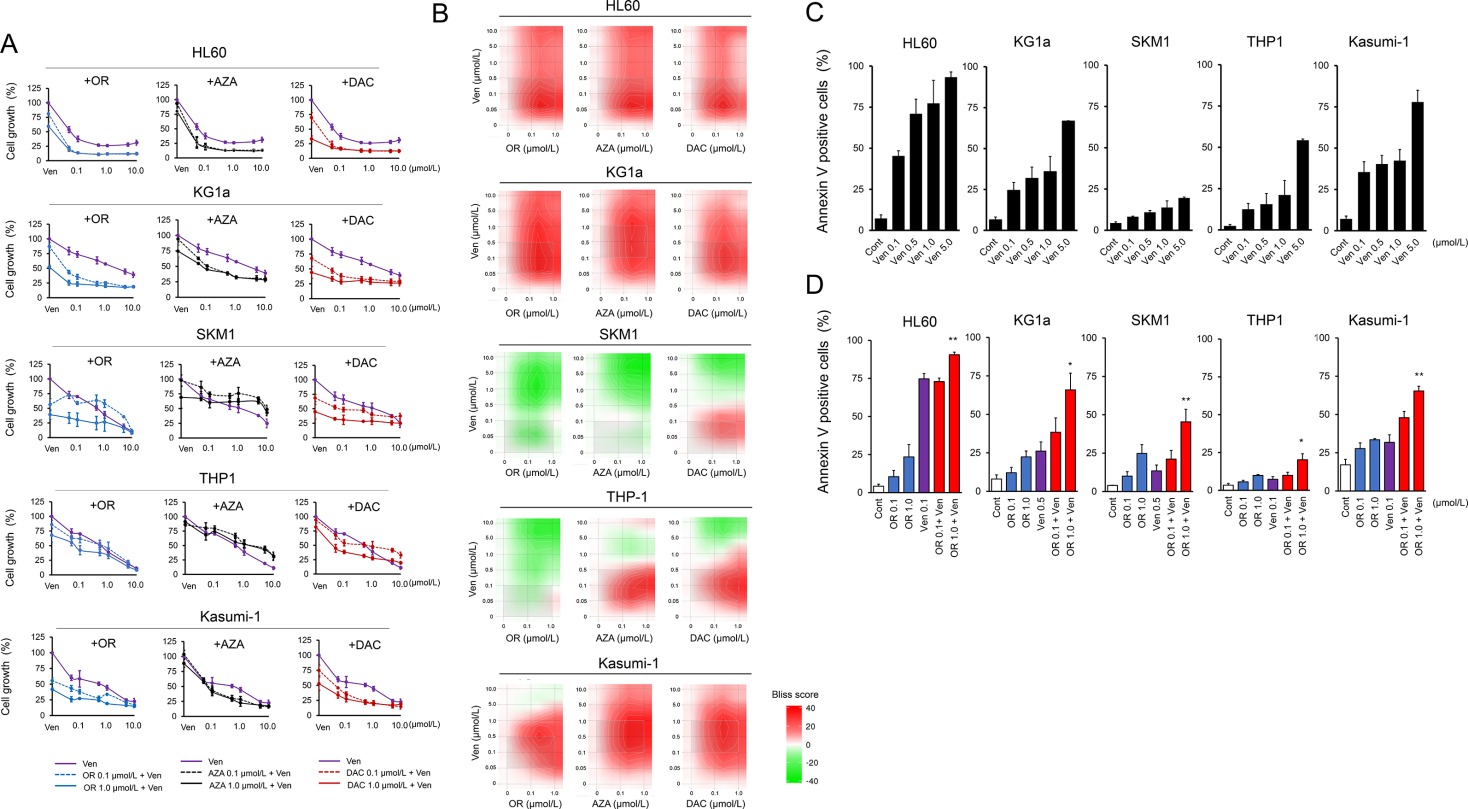
The combination of hypomethylating agents plus Ven shows antileukemia effects against AML. Ven monotherapy inhibited growth of AML cell lines (HL60 and KG1a) in a dose-dependent manner, and addition of 0.1 or 1.0 μmol/L OR21 (OR) exhibited synergistic effects. Cell growth was assessed after 72 hours incubation with each drug (**A**). Two-dimensional synergy maps of each combination treatment in cell growth assays. Synergy maps were generated using SynergyFinder (**B**). Ven monotherapy induced apoptosis in a dose-dependent manner (**C**), and the combination of 0.1 or 1.0 μmol/L OR21 plus Ven (0.1 μmol/L for HL60, THP1, and Kasumi-1; 0.5 μmol/L for KG1a and SKM1) induced apoptosis to a greater extent than Ven monotherapy. Apoptosis was assessed after 72 hours incubation with each drug (**D**). *, *P* < 0.05; **, *P* < 0.01.

Similar to DAC (11), OR21 monotherapy inhibited growth of AML cell lines (HL60, SKM1, THP1, KG1a, and Kasumi-1) in a dose-dependent manner (Supplementary Fig. S1). Ven monotherapy also inhibited growth of AML cell lines in a dose-dependent manner (Fig. 1A). The IC_50_ of HMAs and Ven are shown in Supplementary Table S1. The combination of OR21 plus Ven had synergistic effects on cell growth inhibition of HL60 and KG1a when compared with Ven monotherapy [data based on Bliss score analysis: a Bliss score >10 indicates synergistic effects (ref. 13; Fig. 1A and B; Supplementary Table S2)]. Ven monotherapy also induced apoptosis in a dose-dependent manner (Fig. 1C). Moreover, according to the Bliss score analysis, the combination of Ven and OR21 induced apoptosis at significantly higher rates than Ven monotherapy (Fig. 1D; Supplementary Fig. S2). These results indicate that HMAs have the potential to enhance the antileukemia effects of Ven (15), although the efficacy depends on the cell type.

### MCL-1 Expression in AML Correlates Inversely with Sensitivity to OR21 plus Ven

**FIGURE 2:**
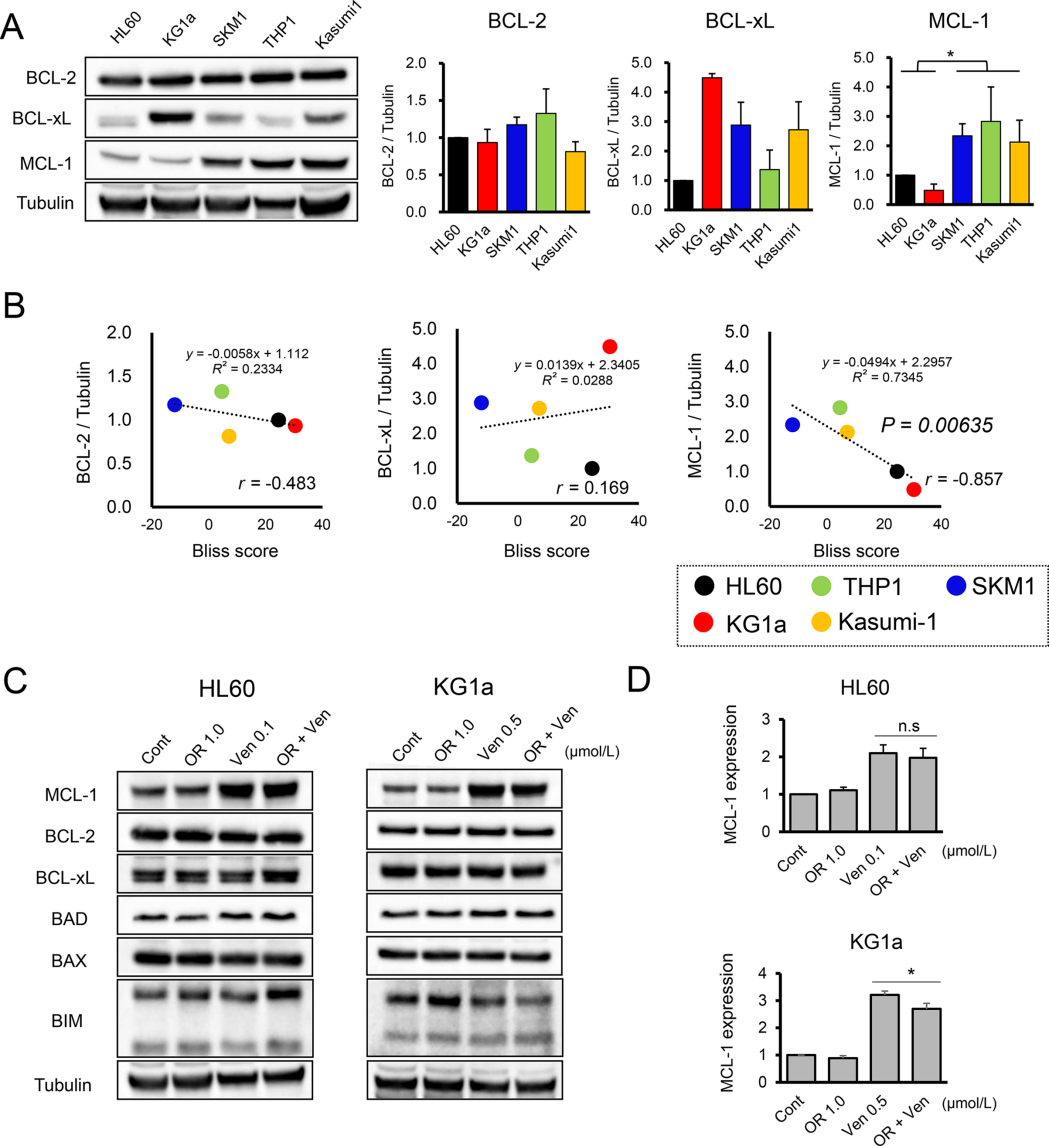
MCL-1 expression correlates inversely with sensitivity of AML cells to OR21 plus Ven. Western blots showing that MCL-1 expression by cell lines (HL60 and KG1a) sensitive to OR21 plus Ven was significantly lower than that of insensitive cell lines (SKM1, THP-1, and Kasumi1). BCL-2 and BCL-xL expression was not associated with sensitivity to OR21 plus Ven (**A**). Correlation between MCL-1 expression and the Bliss score: MCL-1 expression correlates inversely with sensitivity to OR21 plus Ven sensitivity (*r* = −0.857; *P* = 0.00635; **B**). Western blotting of HL60 and KG1a cell lysates after 48 hours incubation with vehicle (Cont), 1.0 μmol/L OR21 (OR), Ven (0.1 μmol/L for HL60, 0.5 μmol/L for KG1a), or OR21 + Ven (OR + Ven). Ven increased expression of MCL-1 by both HL60 (*P =* 0.00218) and KG1a (*P* = 0.0000237) cells, but did not affect expression of other BCL-2 family members (BCL-2; BCL-xL; BAD; BIM (BIM_EL_ and BIM_L_) and BAX; **C**). OR21 plus Ven reduced the Ven-induced increase in MCL-1 expression by KG1a, but not by HL60 (**D**). *, *P* < 0.05; **, *P* < 0.01.

Next, we questioned what determines the sensitivity of AML to HMAs plus Ven. Several studies have reported that the overexpression of antiapoptotic BCL-2 family members (e.g., MCL-1) determines sensitivity to Ven (16). Thus, we assessed the correlation between baseline expression levels of antiapoptotic BCL-2 family members (i.e., BCL-2, BCL-xL, and MCL-1) and the Bliss scores for OR21 plus Ven. We found that the MCL-1 expression in the HL60 and KG1a cell lines, which are sensitive to OR21 plus Ven was significantly lower than that in the OR21 + Ven-insenitive cell lines (i.e., SKM1, THP-1, and Kasumi1). However, BCL-2 and BCL-xL expression did not correlate with sensitivity to OR21 plus Ven (Fig. 2A). Notably, MCL-1 expression correlated inversely with sensitivity to OR21 plus Ven, despite the small number of cell lines tested (*r* = −0.857; *P* = 0.00635; Fig. 2B; Supplementary Table S2). Ven treatment increased the expression of MCL-1 (17) in both the HL60 and KG1a cells, but did not affect the expression of other BCL-2 family members (i.e., BCL-2, BCL-xL, BAD, BIM, and BAX; Fig. 2C). OR21 plus Ven reduced the Ven-induced increase in MCL-1 expression in KG1a but not in HL60 cells (Fig. 2D; Supplementary Fig. S3). Moreover, we investigated changes in the MMP. Ven monotherapy decreased the MMP of HL60 and KG1a cells; however, OR21 did not alter the MMP, either when used as a monotherapy or when used as part of a combination therapy (Supplementary Fig. S4). These results indicate that the synergistic effect of OR21 plus Ven against AML may depend on the baseline expression of MCL-1. In addition, the Ven-mediated increase in MCL-1 levels did not contribute to OR21 plus Ven therapy resistance. Furthermore, OR21 did not change the expression of MCL-1 or other BCL-2 family members, or alter the MMP after combination therapy.

### MCL-1 Expression does not Correlate with Sensitivity of AML to OR21 plus MCL-1 Inhibitors

**FIGURE 3:**
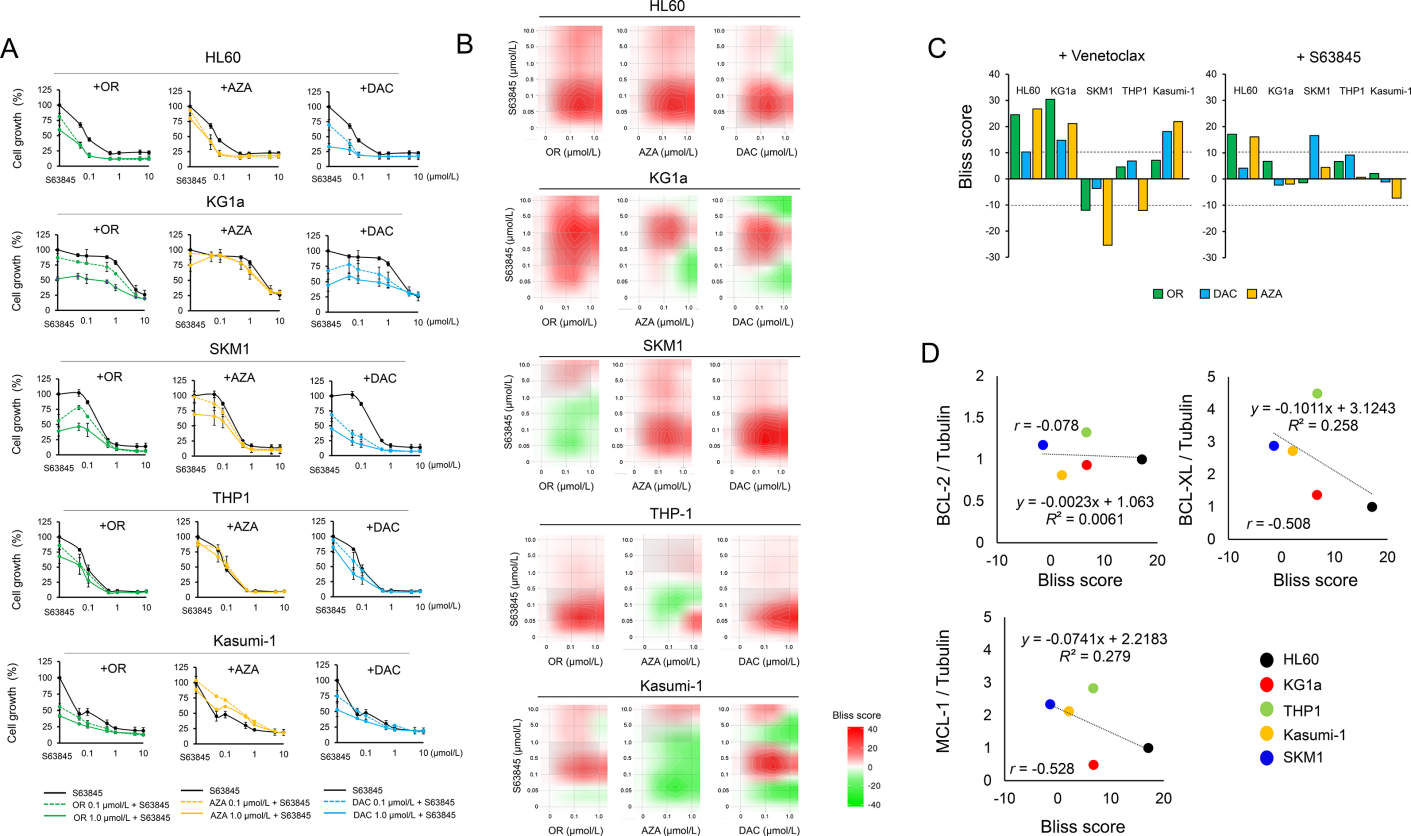
MCL-1 expression does not correlate inversely with sensitivity to OR21 plus the MCL-1 inhibitor S63845. S63845 inhibited cell growth in AML cell lines in a dose-dependent manner, while OR21 plus S63845 did not have a synergistic effect. Cell growth was assessed after a 72-hour incubation with each drug. **A,** Two-dimensional synergy maps of each combination treatment in cell growth assays. Synergy maps were generated using SynergyFinder (**B**). Correlation between MCL-1 expression and the Bliss score: no significant correlation between BCL-2 family protein expression, including MCL-1 expression and the Bliss score, was observed for OR21 plus S63845 (**C**). The comparison of Bliss scores between HMAs plus Ven and HMAs plus S63845 treatment conditions (**D**).

Because MCL-1 overexpression may be involved in resistance to Ven plus HMAs, inhibiting MCL-1 could potentially increase the efficacy of HMAs against AML, especially in cells expressing high MCL-1 levels (18). Therefore, we investigated the combined effects of HMAs plus a selective MCL-1 inhibitor, S63845. We found that S63845 inhibited the growth of AML cell lines in a dose-dependent manner (Fig. 3A). However, OR21 plus S63845 only showed synergistic effects in HL60 but not in KG1a cells, and the Bliss scores were lower than those of the OR21 plus Ven treatment (Fig. 3A–C; Supplementary Table S3). The synergistic effects were not observed in SKM1, THP1, or Kasumi-1 cells, which have relatively high MCL-1 expressions. Furthermore, no significant correlation was observed between BCL-2 family protein expression (including MCL-1), and the Bliss scores of the OR21 plus S63845 therapy (Fig. 3D). These results indicate that S63845 does not overcome the chemoresistance conferred by high MCL-1 expression. To further assess whether MCL-1 levels altered the sensitivity to HMAs plus Ven, we tested the effect of the Ven plus S63845 combination. The cells treated with the combination therapy exhibited significantly higher rate of apoptosis than the monotherapy-exposed cells (Supplementary Fig. S5). Thus, Ven plus HMAs was more effective than the S63845 plus HMAs combination.

### The OR21 plus Ven Combination Significantly Reduced the Expression of the Mitochondrial Homeostatic Protein VAMP7

**FIGURE 4:**
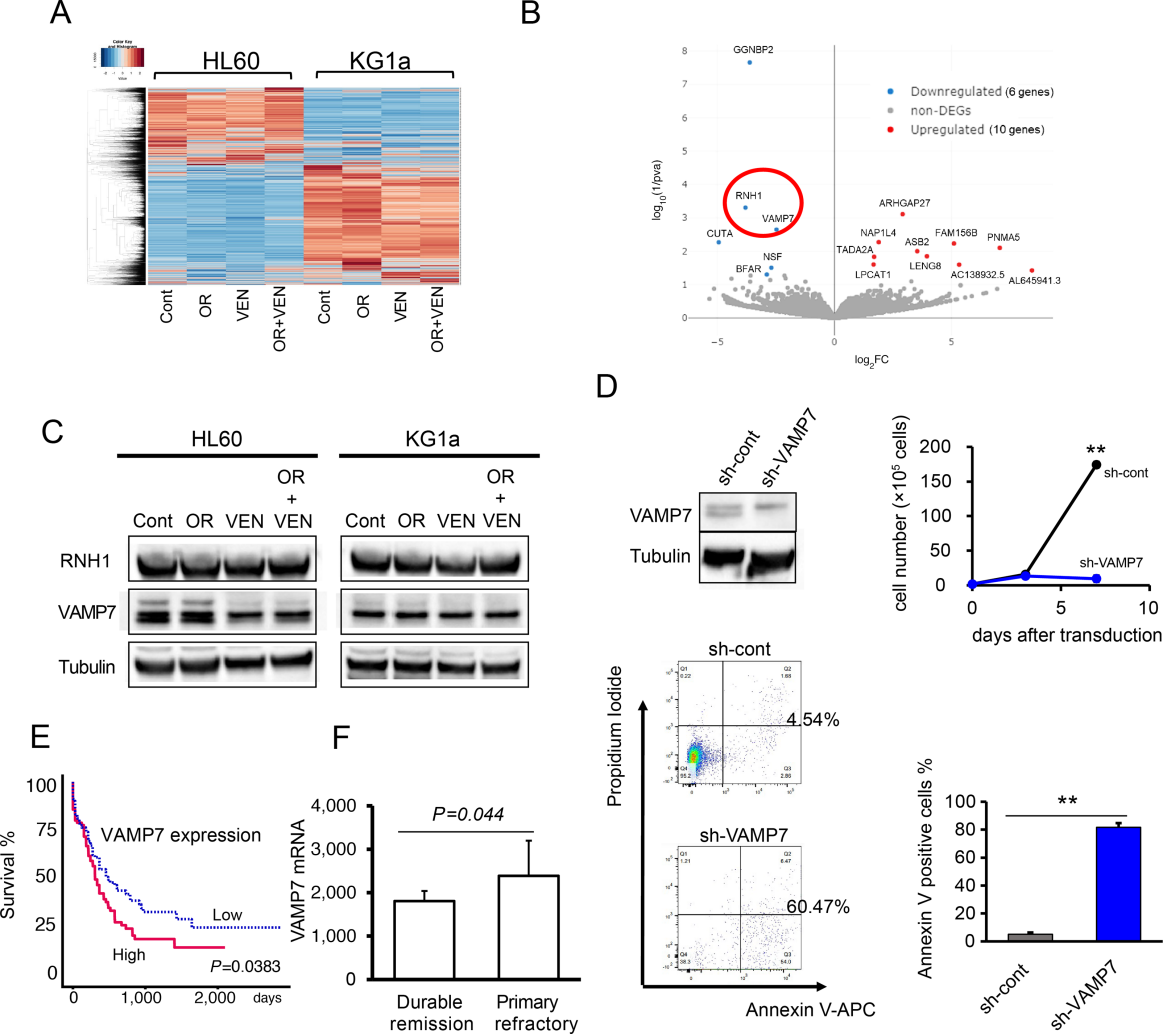
OR21 plus Ven treatment significantly reduces the expression of mitochondrial homeostatic protein VAMP7. Heatmap comparing log_2_ fold changes in gene expression (RNA sequencing) among two OR21 plus Ven-sensitive cell lines (HL60 and KG1a) following a 12-hour treatment; vehicle (Cont), 1.0 μmol/L OR21 (OR), Ven (0.1 μmol/L for HL60, or 0.5 μmol/L for KG1a), and OR21 plus Ven (OR + Ven; **A**). DEG analysis showed that OR21 plus Ven significantly upregulated expression of 10 genes (fold change ≥ 2.0, *P* < 0.05; red dots) and downregulated expression of six genes (fold change < 0.5, *P* < 0.05; blue dots) in both HL60 and KG1a cells compared with Ven monotherapy (**B**). VAMP7 protein levels in HL60 cells decreased 48 hours after exposure to Ven monotherapy or OR21 plus Ven, while RNH1 protein levels remained unaltered in both HL60 cell and KG1a cells (**C**). (The loading controls for two sets of Western blots in Figs. 2C and 4C have been reused). Western blot analysis of HL60 cells transduced with a lentiviral vector expression the *VAMP7*-specific shRNA (sh-VAMP7) or the control lentiviral vector. Ninety-six hours after lentiviral infection of HL60 cells, the knockdown of *VAMP7* reduced cell growth and increased cell apoptosis compared with the control vector (**D**). Representative plots from HL60 cells transduced with the control or sh-VAMP7 vectors. VAMP7 expression level data for in 172 patients with AML were obtained from TCGA (https://tcga-data.nci.nih.gov/tcga/; **E**). *VAMP7* mRNA levels of patients with AML who were treated with Ven plus HMAs or cytarabine (14). Durable remission: *n* = 10, primary refractory: *n* = 15 (**F**).

Having shown that OR21 plus Ven synergistically inhibited the growth of HL60 and KG1a cells, we proceeded to investigate the mechanism by which OR21 promotes Ven-induced antileukemia effects. To this end, we assessed the gene expression profiles of HL60 and KG1a cells following treatment with vehicle, OR21, Ven, or OR21 plus Ven. Sequencing of total RNA exacted from HL60 and KG1a cells was performed 12 hours after exposure to vehicle or drugs (Fig. 4A). The DEG analysis of HL60 and KG1a cells showed that OR21 plus Ven significantly upregulated the expression of 10 genes (fold change ≥2.0, *P* < 0.05; red dots) and downregulated the expression of six genes (fold change < 0.5, *P* < 0.05; blue dots) when compared with Ven monotherapy; the transcripts per million values for the 16 DEGs are shown in Supplementary Tables S4 and S5. Among these, *RNH1*, which plays a protective role in cellular redox homeostasis by catalyzing redox reactions (19), and *VAMP7*, a member of the soluble N-ethylmaleimide–sensitive factor attachment protein receptor (SNARE) family, which is involved in the autophagic maintenance of mitochondrial homeostasis (20), were significantly downregulated following exposure to OR21 plus Ven (Fig. 4B). We also found that VAMP7 protein levels decreased in HL60 cells after exposure to Ven monotherapy or OR21 plus Ven, while the RNH1 protein levels were unaltered in both HL60 and KG1a cells under the same treatment conditions (Fig. 4C). To investigate the impact of VAMP7 knockdown on AML cells, we transduced HL60 cells with a lentiviral vector encoding a *VAMP7*-targeted short hairpin (sh) RNA. *VAMP7* knockdown attenuated cell growth and increased cell apoptosis relative to the control 96 hours after infection (Fig. 4D).

We further evaluated the prognostic impact of *VAMP7* expression using a dataset from The Cancer Genome Atlas (TCGA; https://tcga-data.nci.nih.gov/tcga/) which included 172 patients with AML. Lower *VAMP7* mRNA predicted favorable survival outcomes in patients with AML (Fig. 4E). Furthermore, lower *VAMP7* mRNA levels were associated with higher Ven plus HMAs or cytarabine responses in patients with AML (Fig. 4F; ref. 14). Because OR21 plus Ven reduced VAMP7 expression and induced the apoptosis of AML cells (Fig. 4D), OR21 plus Ven could be more effective at treating patients with AML with lower VAMP7 expression. These results suggested that OR21 plus Ven lowered VAMP7 expression which may be associated with more favorable survival outcomes or treatment responses in patients with AML.

### OR21 plus Ven Triggers Significant Accumulation of ROS and Triggers ROS-induced Apoptosis

**FIGURE 5:**
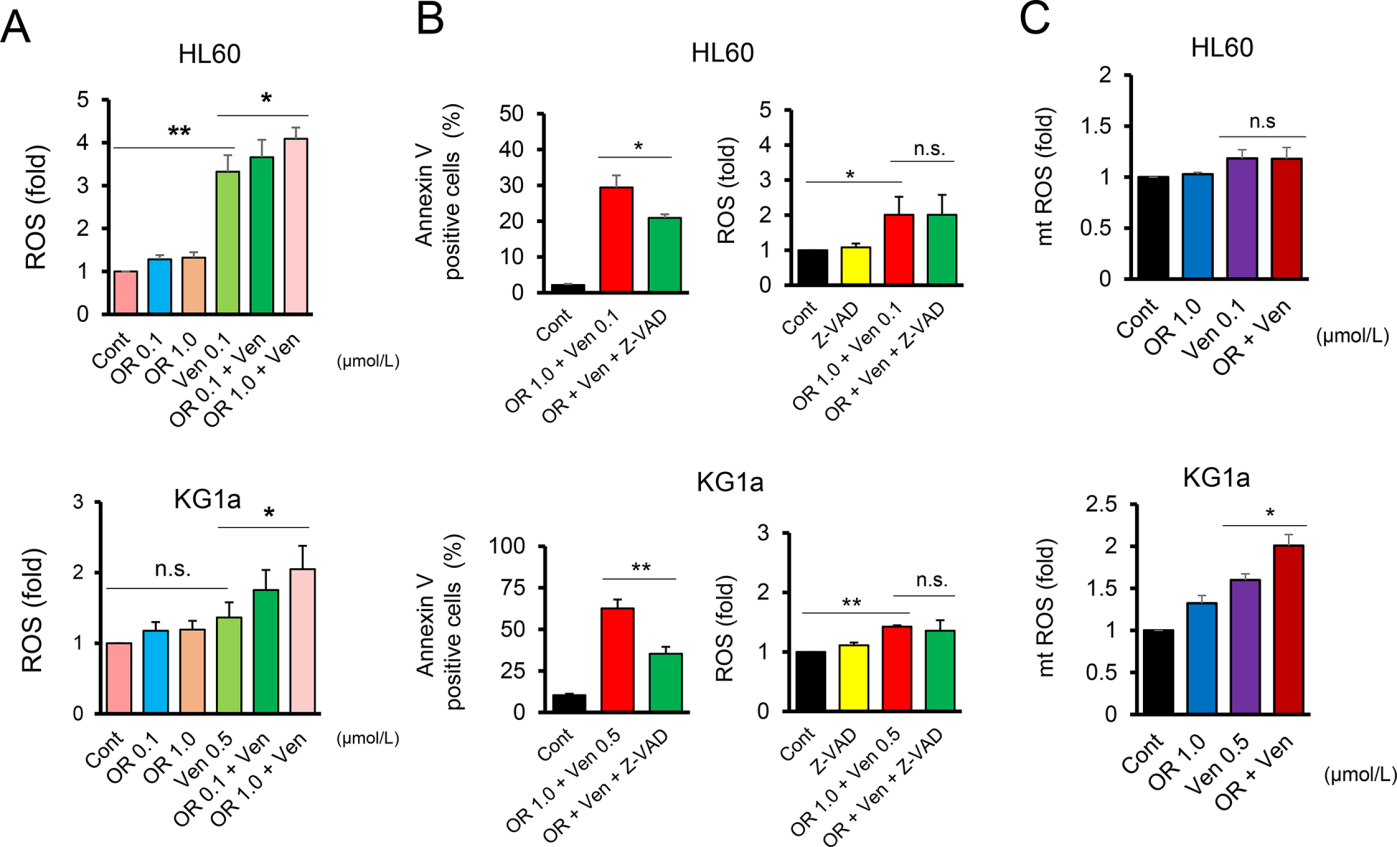
OR21 increases the production of ROS. Flow cytometry analysis shows that OR21 monotherapy does not increase ROS accumulation in HL60 and KG1a cells, whereas Ven monotherapy significantly increases ROS accumulation; OR21 plus Ven increases ROS accumulation even further (**A**). Pretreatment with Z-VAD-FMK (Z-VAD), a pan caspase inhibitor, reduces OR21 plus Ven-induced apoptosis to a greater extent than OR21 plus Ven, but does not affect ROS accumulation. Apoptosis and ROS were detected after a 24-hour incubation with OR21 plus Ven (**B**). Quantification of mtROS levels. OR21 plus Ven significantly increased mtROS accumulation compared with Ven monotherapy in KG1a cells (**C**). *, *P* < 0.05; **, *P* < 0.01.

Autophagy prevents DNA damage by removing oxidized biomolecules (21). We therefore examined the effect of monotherapy or combination therapy on the production of ROS. OR21 monotherapy did not increase ROS accumulation in HL60 and KG1a cells, whereas Ven monotherapy increased ROS accumulation significantly; this accumulation was even greater after treatment with OR21 plus Ven (Fig. 5A). Similar results were observed after treatment with AZA plus Ven, which also promoted ROS accumulation in HL60 and KG1a (although this was not statistically significant in KG1a cells), when compared with Ven monotherapy (Supplementary Fig. S6). This indicates that OR21 and AZA may employ similar antileukemia mechanisms because they are both DNA (cytosine-5)-methyltransferase 1 (DNMT1) inhibitors. Treatment with Z-VAD-FMK, a pan caspase inhibitor, prior to exposure to OR21 plus Ven decreased cell apoptosis in HL60 and KG1a cells compared with OR21 plus Ven, but did not affect ROS accumulation (Fig. 5B). We also examined mtROS levels. OR21 plus Ven significantly increased mtROS accumulation compared with Ven monotherapy in KG1a cells (Fig. 5C). These results indicate that ROS accumulation after treatment with OR21 plus Ven is not due to apoptosis; rather, OR21 plus Ven triggers ROS accumulation, which then induces apoptosis of AML cells.

### OR21 Increases ROS Levels by Potentially Suppressing Ven-induced Mitophagy

Gene expression profiling revealed that OR21 significantly downregulated *VAMP7*, a SNARE protein that plays an important role in autophagic maintenance during mitochondrial homeostasis; therefore, we hypothesized that OR21 may affect mitophagy/autophagy pathways. Although Ven did not alter the levels of the autophagy marker LC3B and only slightly reduced the levels of p62 (Supplementary Fig. S7A), it induced mitophagy. Ven-induced mitophagy was attenuated by the addition of OR21 (Supplementary Fig. S7B and S7C). Additional treatment of HL60 cells with Rapa, an inducer of mitophagy/autophagy, significantly decreased OR21 plus Ven-induced ROS accumulation (Supplementary Fig. S7D). Furthermore, pretreatment with Rapa or PMI significantly decreased cell apoptosis when compared with OR21 plus Ven (Supplementary Fig. S7E). These results indicate that mitophagy protects AML cells from ROS-induced apoptosis, and that OR21 inhibits the mitophagy pathway [which is involved in clearance of damaged mitochondria to maintain mitochondrial homeostasis (22)], thereby promoting apoptosis via increased ROS accumulation following Ven-induced mitochondrial damage.

### OR21 plus Ven Exhibit Antitumor Effects in a Xenograft Mouse Model

**FIGURE 6:**
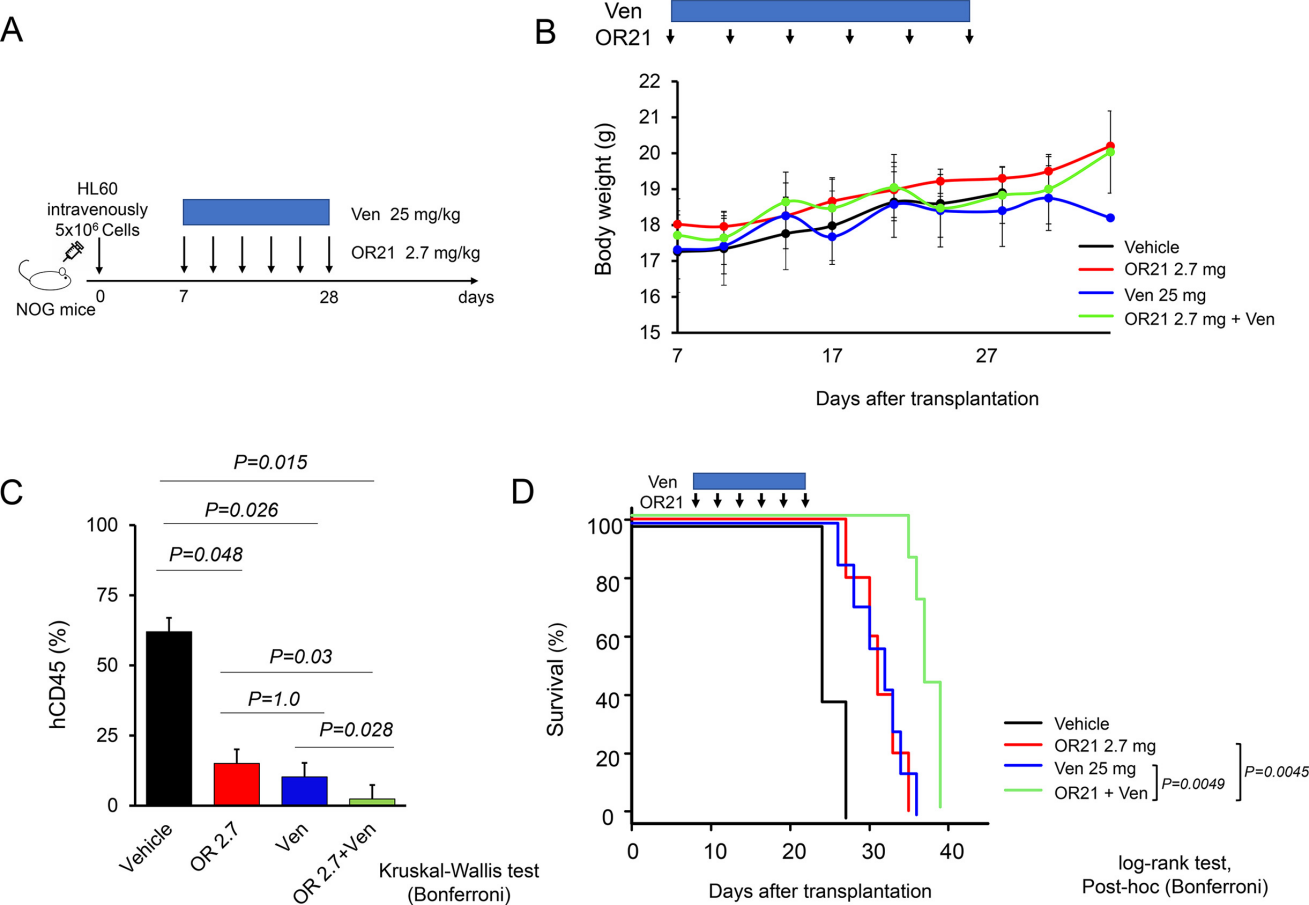
OR21 plus Ven exhibit antitumor effects against HL60 cells in a xenograft mouse model. Experimental schema showing the HL60 cell xenograft experiments in NOG mice. NOG mice were injected intravenously with HL60 cells. Mice were then treated with OR21 (2.7 mg/kg), Ven (25 mg/kg), or OR21 plus Ven (OR21 2.7 mg/kg + Ven 25 mg/kg) for 21 days, starting on day 7 posttransplantation (**A**). Average mouse body weight (error bars represent the SD in each treatment group) was measured twice per week. No weight loss or severe toxicity were observed in any group during the treatment period (**B**). Flow cytometry analysis shows that the tumor burden of human CD45^+^ (hCD45) leukemic cells in the peripheral blood on day 28. The percentage of human CD45^+^ cells in the peripheral blood of OR21 plus Ven-treated mice was significantly lower than that in OR21 or Ven-treated mice (**C**). Kaplan–Meier analysis shows that OR21 plus Ven-treated mice survive for significantly longer than OR21 or Ven-treated mice (*P* = 0.0049 vs. Ven; *P =* 0.0045 vs. OR21; **D**).

Finally, to examine the antileukemia effects of OR21 plus Ven *in vivo*, we injected NOG mice intravenously with HL60 cells. Mice were then treated with vehicle (1% DMSO and dissolution of VEN), OR21 (2.7 mg/kg), Ven (25 mg/kg), or OR21 plus Ven (OR21 2.7 mg/kg + Ven 25 mg/kg) for 21 days, starting on day 7 posttransplantation (Fig. 6A). No weight loss or severe toxicity was observed in any group during the treatment period (Fig. 6B). On day 28, the percentage of human CD45-positive cells in the peripheral blood of OR21 plus Ven-treated mice was significantly lower than that in OR21- or Ven-treated mice (Fig. 6C). OR21 plus Ven-treated mice also survived for significantly longer than OR21- or Ven-treated mice (*P* = 0.0049 vs. Ven; *P* = 0.0045 vs. OR21; Fig. 6D). We then examined the safety and efficacy of higher doses of the OR21/Ven combination. Mice were treated with vehicle (1% DMSO and dissolution of VEN), OR21 (5.4 mg/kg), Ven (25 mg/kg), or OR21 plus Ven (OR21 5.4 mg/kg + Ven 25 mg/kg) for 21 days, starting on day 7 posttransplantation (Supplementary Fig. S8A). No weight loss or severe toxicity was observed in any group during the treatment period (Supplementary Fig. S8B). No additional antitumor effects were observed in OR21 (5.4 mg/kg) plus Ven (25 mg/kg)-treated mice compared with OR21 (5.4 mg/kg)- or Ven (25 mg/kg)-treated mice (Supplementary Fig. S8C and S8D).

**FIGURE 7:**
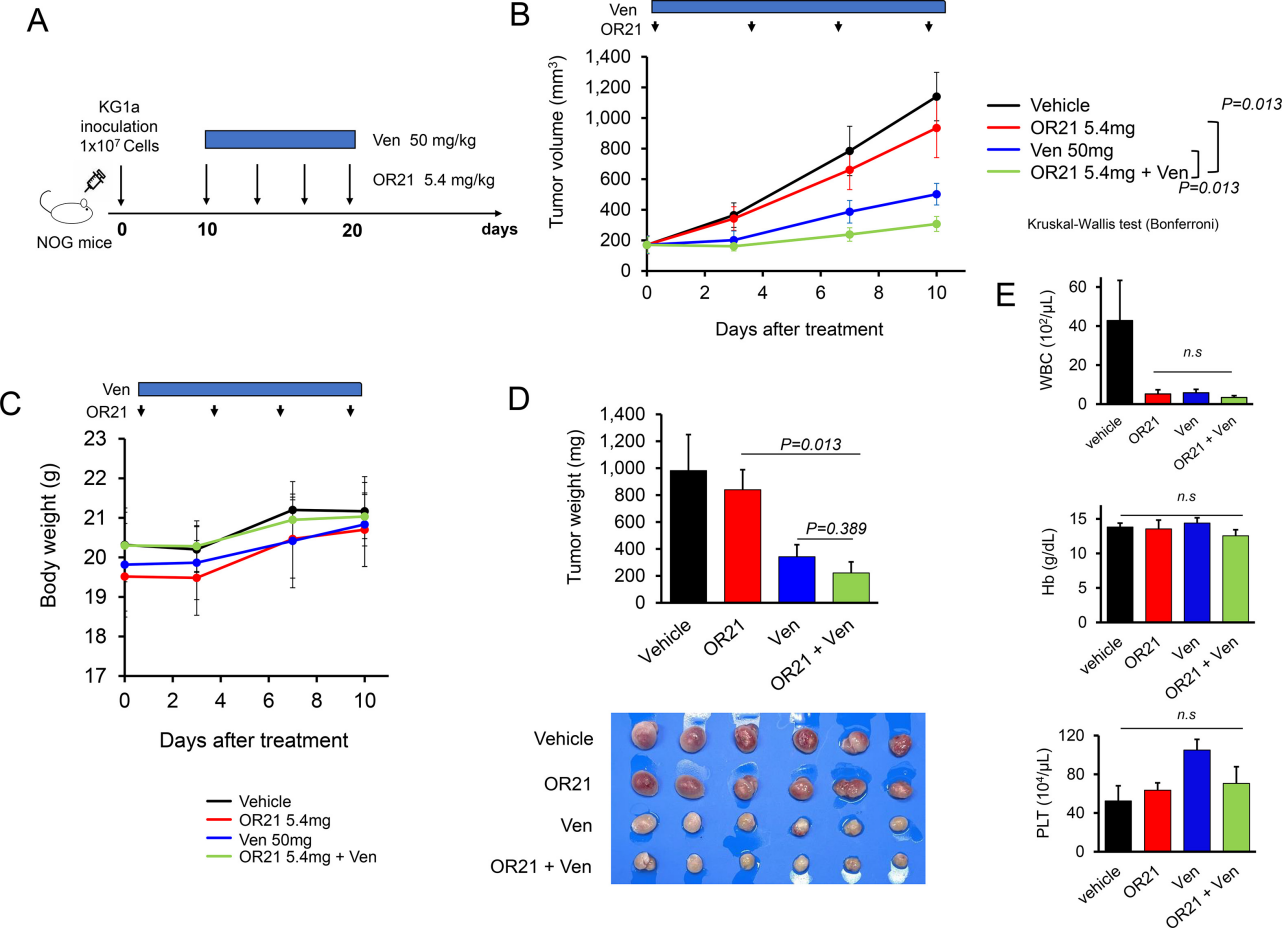
OR21 plus Ven exhibit antitumor effects against KG1a cells in a xenograft mouse model. Experimental schema showing the KG1a cell xenograft experiments in NOG mice. NOG mice were subcutaneously inoculated with KG1a cells. Mice were then treated with OR21 (5.4 mg/kg), Ven (50 mg/kg), or OR21 plus Ven (OR21 5.4 mg/kg + Ven 50 mg/kg) for 10 days, starting on day 10 postinoculation (**A**). Mean tumor volume during treatment. OR21 plus Ven significantly suppressed tumor growth on day 10 after treatment initiation (vs. Ven: *P =* 0.013; **B**). Average mouse body weights were recorded twice per week. No weight loss or severe toxicity was observed in any group during the treatment period (**C**). Mean tumor weights and images of the xenograft tumors isolated from all mice are shown. OR21 plus Ven suppressed tumor growth (**D**). Concentration of white blood cells (WBC), hemoglobin (Hg), and platelets (PLT) on day 21 after inoculation. OR21 plus Ven was not associated with adverse neutropenia, anemia, or thrombocytopenia (**E**).

We also examined another xenograft model using KG1a cells. KG1a cells were chosen because they were the most Ven-resistant (according to the IC_50_ values) among all the AML cell lines tested. We subcutaneously inoculated KG1a cells into NOG mice and commenced treatment with vehicle (1% DMSO and dissolution of Ven), OR21 (5.4 mg/kg), Ven (50 mg/kg), or OR21 plus Ven (OR21 5.4 mg/kg + Ven 50 mg/kg) on day 10 after inoculation for a total of 10 days (Fig. 7A). OR21 plus Ven significantly suppressed tumor growth compared with Ven monotherapy (*P =* 0.013) or OR21 monotherapy (*P* = 0.013; Fig. 7B), without severe toxicity (Fig. 7C). OR21 plus Ven also decreased tumor weight after the completion of treatment (Fig. 7D). Furthermore, the addition of OR21 to Ven did not increase hematotoxicity (Fig. 7E).

These results indicate that an appropriate dose of OR21 plus Ven has stronger anti-leukemia effects than OR21 or Ven monotherapy, without any increase in toxicity.

## Discussion

Here, we show that combination treatment with OR21 plus Ven has antileukemia effects. Ven plus HMAs such as AZA or DAC is now the standard treatment strategy for patients with transplant-ineligible AML (3–5). However, AZA or DAC are easily degraded by CDA, thereby limiting their bioavailability after oral administration. Therefore, they must be administered intravenously or subcutaneously. Although orally bioavailable HMAs (i.e., CC-486 and ASTX727) have been approved, their safety, efficacy, and bioavailability require improvement.

Clinical trials designed to investigate the safety, efficacy, and pharmacokinetics of a combination of oral HMAs plus Ven are now ongoing (NCT04102020 and NCT04746235). Previously, we developed OR21, which is resistant to degradation by CDA. OR21 shows favorable oral bioavailability and has antileukemia effects similar to those of AZA or DAC (but with a better safety profile than DAC) at the same area under the plasma drug concentration–time curve (AUC); this may be because it achieves a lower peak plasma concentration than DAC (10–12). OR21 also overcomes AZA resistance of AML (11). Furthermore, OR21 is a single compound; thus, OR21 has enormous advantages with respect to ease of production and quality control. Hence, a combination of OR21 and Ven could be administered orally, which would potentially improve patient quality of life (23). The sensitivity of AML to OR21 plus Ven may depend on MCL-1 expression as shown in Fig. 2B [similarly to the sensitivity of AML to AZA plus Ven (24)]. However, because of the small numbers of AML cell lines we tested in the current study, these correlations need to be validated in large patient cohorts or cell lines panels.

**FIGURE 8:**
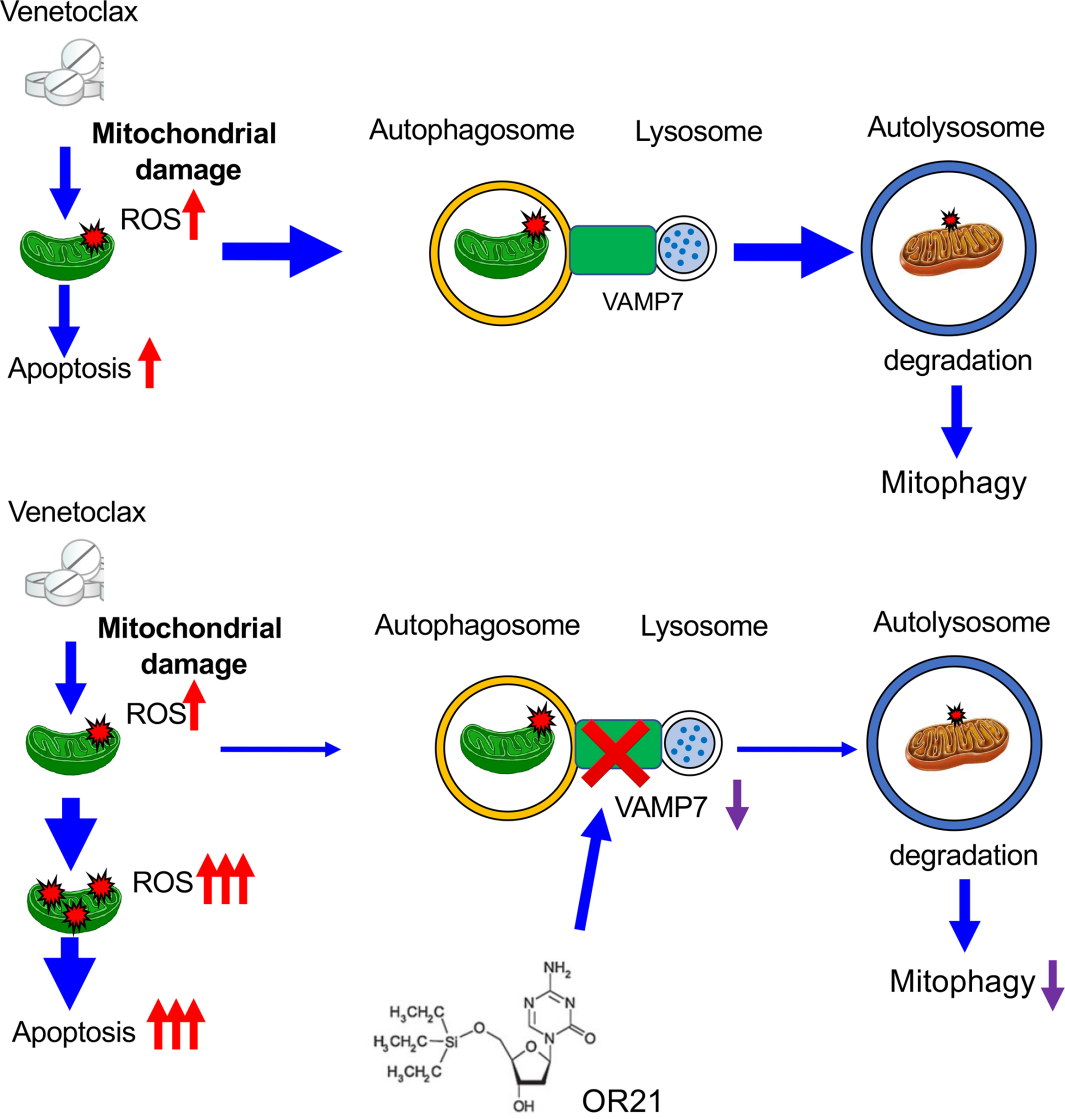
Graphical schema showing the putative mechanism underlying the effects of combination therapy with OR21 and Ven. Putative mechanism underlying the antileukemia effects of OR21 plus Ven. Ven induces mitochondrial damage via accumulation of ROS, which triggers mitophagy to remove damaged mitochondria and reduce ROS accumulation (a response that protects against ROS-mediated apoptosis). OR21 inhibits the mitophagy pathway, meaning that mitochondria damaged by Ven are not removed, leading to ROS accumulation and promotion of Ven-induced cell apoptosis.

Ven inhibits mitochondrial respiration, BCL-2 (25), while also triggering ROS accumulation (26). Excessive ROS induces mitochondrial stress responses, leading to the activation of cell death pathways. Mitophagy ensures the selective removal of damaged mitochondria (27) to maintain mitochondrial homeostasis. Our results suggest that OR21 epigenetically downregulated expression of VAMP7 (28, 29), thereby inhibiting mitophagy. After inhibition of mitophagy, mitochondria damaged by Ven cannot be removed, leading to the accumulation of ROS and the activation of cell apoptosis pathways. Unfortunately, we did not fully explain the antileukemia mechanism of OR21 plus Ven with regards to the inhibition of the autophagy/mitophagy pathway. However, we showed that the knockdown of *VAMP7* markedly inhibited the growth and promoted the apoptosis of AML cells. Because *VAMP7* is involved in the maintenance of mitochondrial homeostasis (20), its downregulation may induce cytotoxicity. Hence, the inhibition of VAMP7 expression may directly contribute to the antileukemia effects of the OR21 plus Ven combination therapy (Fig. 8).

*In vivo*, Ven plus OR21 exhibited higher efficacy than Ven or OR21 monotherapy, without an increase in toxicity. Moreover, we previously reported that that same AUC dose of DAC (as OR21) did not exert an antileukemia effect but did exhibit toxicity in the HL60 xenograft mice model (10, 11). We therefore surmised that the combination of DAC and Ven might exhibit more toxicity than DAC monotherapy. Thus, we did not test the DAC plus Ven *in vivo*. Moreover, OR21 is an effective treatment for AZA-resistant leukemia *in vi*vo (11, 30). Hence, the combination of Ven plus OR21 may benefit patients with AML, including AZA-resistant AML, thereby replacing the standard therapy. We are currently conducting a phase I clinical trial of OR21 (Japan Registry Clinical Trials number, jRCT2071220035) to evaluate its safety, tolerability, and pharmacokinetics in patients with high-risk myelodysplastic syndromes.

## Supplementary Material

Figure S1 Figure S1. Cell growth inhibition by hypomethylating agent monotherapy in acute myeloid leikemia (AML).

Figure S2 Figure S2. 3D synergy maps of each combination treatment (OR21 plus venetoclax) in apoptosis assays.

Figure S3 Figure S3. BCL2 and BCL-xL levels in HL60 and KG1a following Vehicle (Cont), 1.0 mM of OR21 (OR1), 0.1 mM (HL60) or 0.5 mM (KG1a) of venetoclax and OR21 and venetoclax (OR+Ven) combination treatment. n.s. indicated not significant.

Figure S4 Figure S4. HL and KG1a cells were exposed to vehicle (Cont), 1.0 mM of OR21 (OR 1), 0.1 μM (HL60) or 0.5 μM (KG1a) of venetoclax (Ven) and OR21 plus venetoclax (OR+Ven). Then mitochondrial membrane potential (MMP) was measured by flow cytometry. Venetoclax decreased MMP levels in HL60 and KG1a, while OR21 did not affect MMP levels in both monotherapy and combination. *p<0.05. n.s. indicated not significant.

Figure S5 Figure S5. The combination effect of venetoclax (Ven) and S63845 (S6) in acute myeloid leukemia.

Figure S6 Figure S6. Flowcytometric analysis for ROS detection following 48 h vehicle treated (cont), 0.1 μM of azacitidine treated (A 0.1), 1.0 μM of azacitidine treated (A 1.0), 0.1 μM of venetoclax treated (V 0.1), 0.5 μM of venetoclax treated (V 0.5), 0.1 μM of azacitidine and 0.1 μM of venetoclax (A 0.1+V 0.1), 1.0 μM of azacitidine and 0.1 μM of venetoclax (A 1.0 + V 0.1), 0.1 μM of azacitidine and 0.5 μM of venetoclax (A 0.1 + V 0.5) and 1.0 μM of azacitidine and 0.5 μM of venetoclax (A 1.0 + V 0.5).

Figure S7 Figure S7. OR21 increases ROS levels by suppressing Ven-induced mitophagy

Figure S8 Figure S8. Schedule of HL60 cell xenograft experiments using NOG mice. NOG mice were injected intravenously with HL60 cells. Mice were then treated with OR21 (5.4 mg/kg), Ven (25 mg/kg), and OR21 plus Ven (OR21+Ven) for 21 days since day 7 after transplantation (A). Average mouse body weights (error bars represent standard deviation) for the treatment groups were measured two times per weeks. No weight loss nor severe toxicity were observed in any group during the treatment period (B). Flow cytometric analysis showed tumor burden of human CD45+ (hCD45) leukemic cells in peripheral blood on day 28. The proportion of human CD45-positive cells in the peripheral blood of OR21 plus Ven-treated mice was not lower than that in OR21 or Ven-treated mice (C). Kaplan–Meier analysis showed OR21 plus Ven-treated mice did not exhibit prolonged survival compared to OR21 or Ven-treated mice (p = 0.308 vs. Ven; p = 1.0 vs. OR21; D).

Table TS1 IC50 in acute myeloid leukemia cell lines (µM)

Table TS2 The Bliss score in combination of venetoclax

Table TS3 The Bliss score in combination of S63845

Table TS4 The transcripts per million (TPM) values of six downregulated genes

Table TS5 The transcripts per million (TPM) values of ten upregulated genes
