# Supplementary material for: Combination of a New Oral Demethylating Agent, OR2100, and Venetoclax for Treatment of Acute Myeloid Leukemia
Source: Cancer Res Commun. 2023 Feb 21;3(2):297–308. doi: 10.1158/2767-9764.CRC-22-0259 (PMC9973401; doi:10.1158/2767-9764.CRC-22-0259)
Supplement: Figure S1 — Cell growth inhibition by hypomethylating agent monotherapy in acute myeloid leikemia (AML). [file crc-22-0259-s01.pdf]

Figure S1

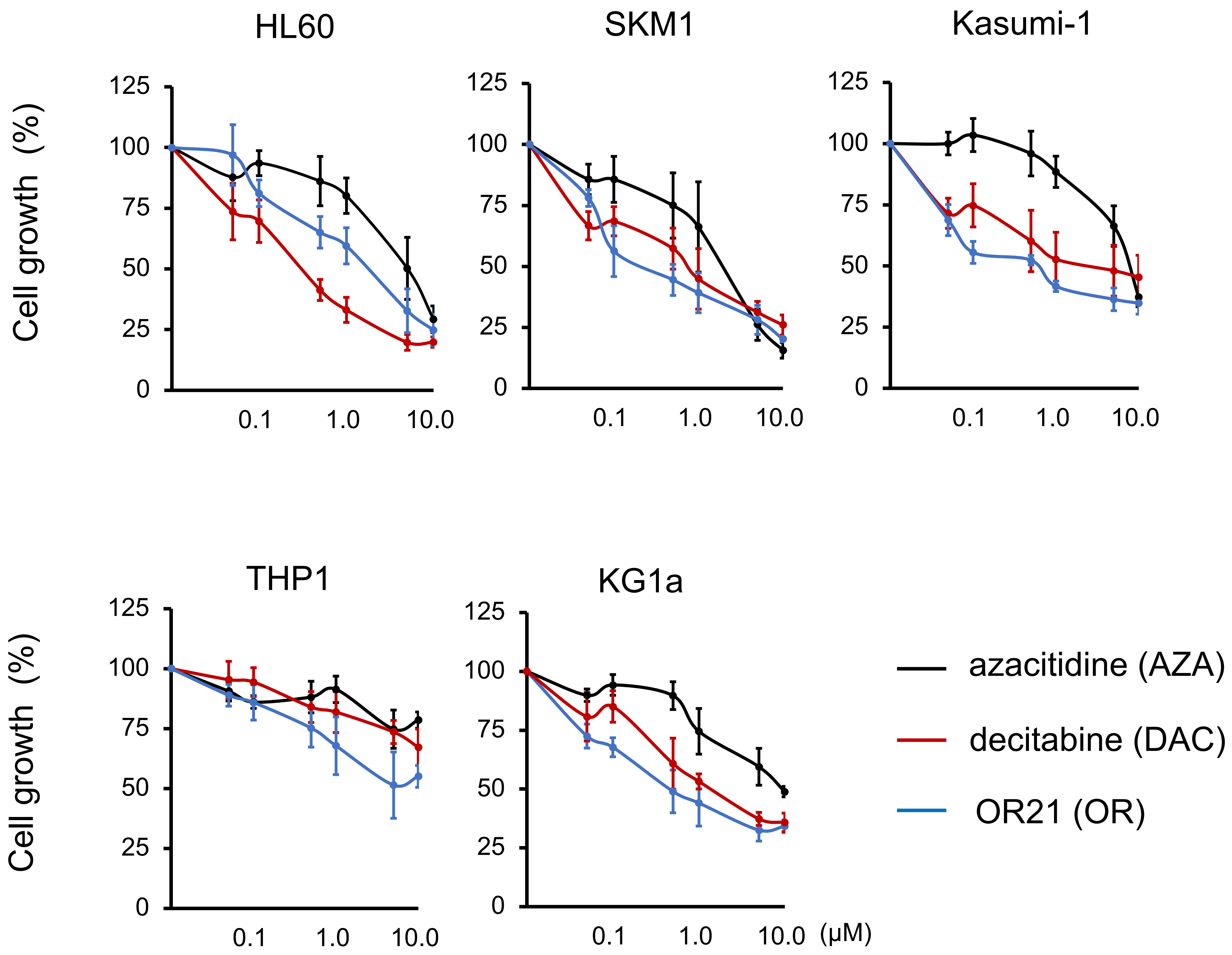

Figure S1. Cell growth inhibition by hypomethylating agent monotherapy in acute myeloid leikemia (AML)

OR21 monotherapy inhibited growth of AML cell lines (HL60, SKM1, THP1, KG1a, and Kasumi-1) in a dose-dependent manner (similar to decitabine) after 72 h incubation.
