# Supplementary material for: Combination of a New Oral Demethylating Agent, OR2100, and Venetoclax for Treatment of Acute Myeloid Leukemia
Source: Cancer Res Commun. 2023 Feb 21;3(2):297–308. doi: 10.1158/2767-9764.CRC-22-0259 (PMC9973401; doi:10.1158/2767-9764.CRC-22-0259)
Supplement: Figure S2 — 3D synergy maps of each combination treatment (OR21 plus venetoclax) in apoptosis assays. [file crc-22-0259-s02.pdf]

Figure S2

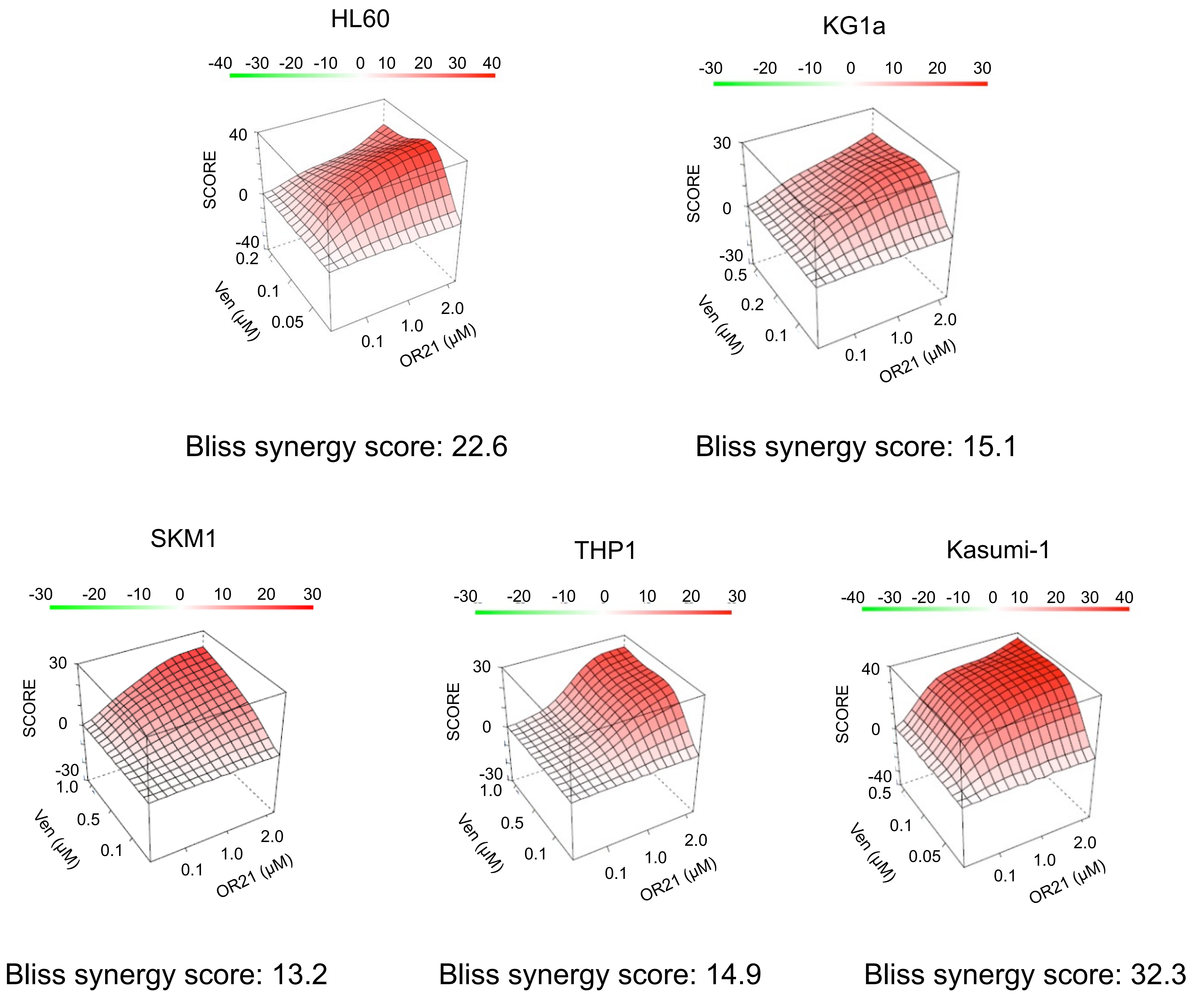

Figure S2. 3D synergy maps of each combination treatment (OR21 plus venetoclax) in apoptosis assays.
