# Supplementary material for: Combination of a New Oral Demethylating Agent, OR2100, and Venetoclax for Treatment of Acute Myeloid Leukemia
Source: Cancer Res Commun. 2023 Feb 21;3(2):297–308. doi: 10.1158/2767-9764.CRC-22-0259 (PMC9973401; doi:10.1158/2767-9764.CRC-22-0259)
Supplement: Figure S3 — BCL2 and BCL-xL levels in HL60 and KG1a following Vehicle (Cont), 1.0 mM of OR21 (OR1), 0.1 mM (HL60) or 0.5 mM (KG1a) of venetoclax and OR21 and venetoclax (OR+Ven) combination treatment. n.s. indicated not significant. [file crc-22-0259-s03.pdf]

Figure S3

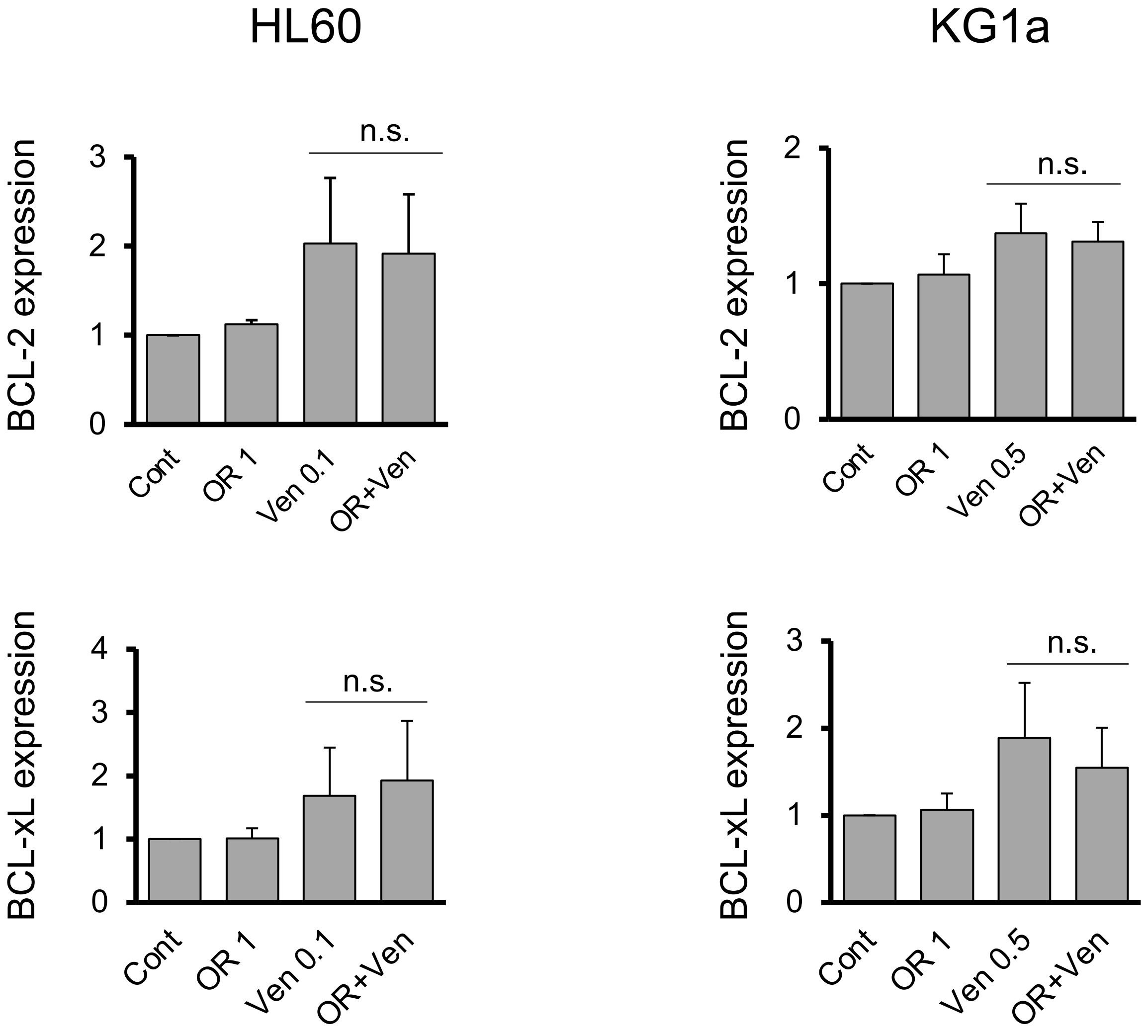

Figure S3. BCL2 and BCL-xL levels in HL60 and KG1a following Vehicle (Cont), 1.0 μM of OR21 (OR1), 0.1 μM (HL60) or 0.5 μM (KG1a) of venetoclax and OR21 and venetoclax (OR+Ven) combination treatment. n.s. indicated not significant.
