# Supplementary material for: Combination of a New Oral Demethylating Agent, OR2100, and Venetoclax for Treatment of Acute Myeloid Leukemia
Source: Cancer Res Commun. 2023 Feb 21;3(2):297–308. doi: 10.1158/2767-9764.CRC-22-0259 (PMC9973401; doi:10.1158/2767-9764.CRC-22-0259)
Supplement: Figure S4 — HL and KG1a cells were exposed to vehicle (Cont), 1.0 mM of OR21 (OR 1), 0.1 μM (HL60) or 0.5 μM (KG1a) of venetoclax (Ven) and OR21 plus venetoclax (OR+Ven). Then mitochondrial membrane potential (MMP) was measured by flow cytometry. Venetoclax decreased MMP levels in HL60 and KG1a, while OR21 did not affect MMP levels in both monotherapy and combination. *p<0.05. n.s. indicated not significant. [file crc-22-0259-s04.pdf]

Figure S4

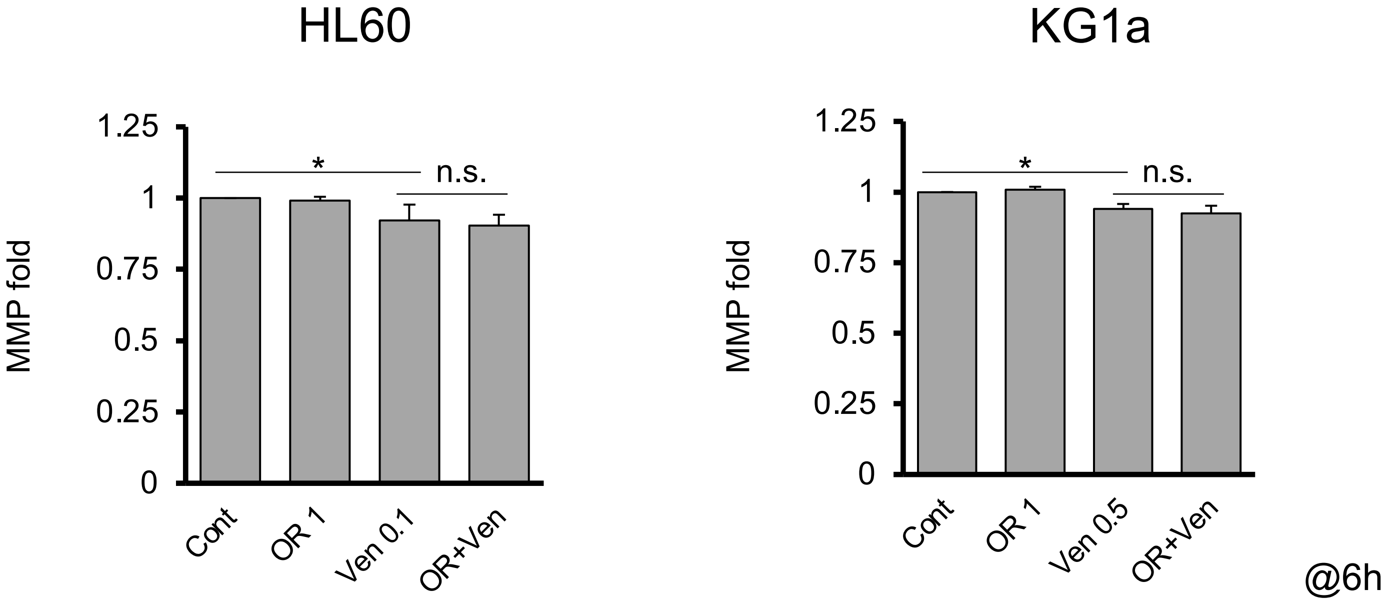

Figure S4. HL and KG1a cells were exposed to vehicle (Cont), 1.0 μM of OR21 (OR 1), 0.1 μM (HL60) or 0.5 μM (KG1a) of venetoclax (Ven) and OR21 plus venetoclax (OR+Ven). Then mitochondrial membrane potential (MMP) was measured by flow cytometry. Venetoclax decreased MMP levels in HL60 and KG1a, while OR21 did not affect MMP levels in both monotherapy and combination. *$p<0.05$. n.s. indicated not significant.
