# Supplementary material for: Combination of a New Oral Demethylating Agent, OR2100, and Venetoclax for Treatment of Acute Myeloid Leukemia
Source: Cancer Res Commun. 2023 Feb 21;3(2):297–308. doi: 10.1158/2767-9764.CRC-22-0259 (PMC9973401; doi:10.1158/2767-9764.CRC-22-0259)
Supplement: Figure S5 — The combination effect of venetoclax (Ven) and S63845 (S6) in acute myeloid leukemia. [file crc-22-0259-s05.pdf]

Figure S5

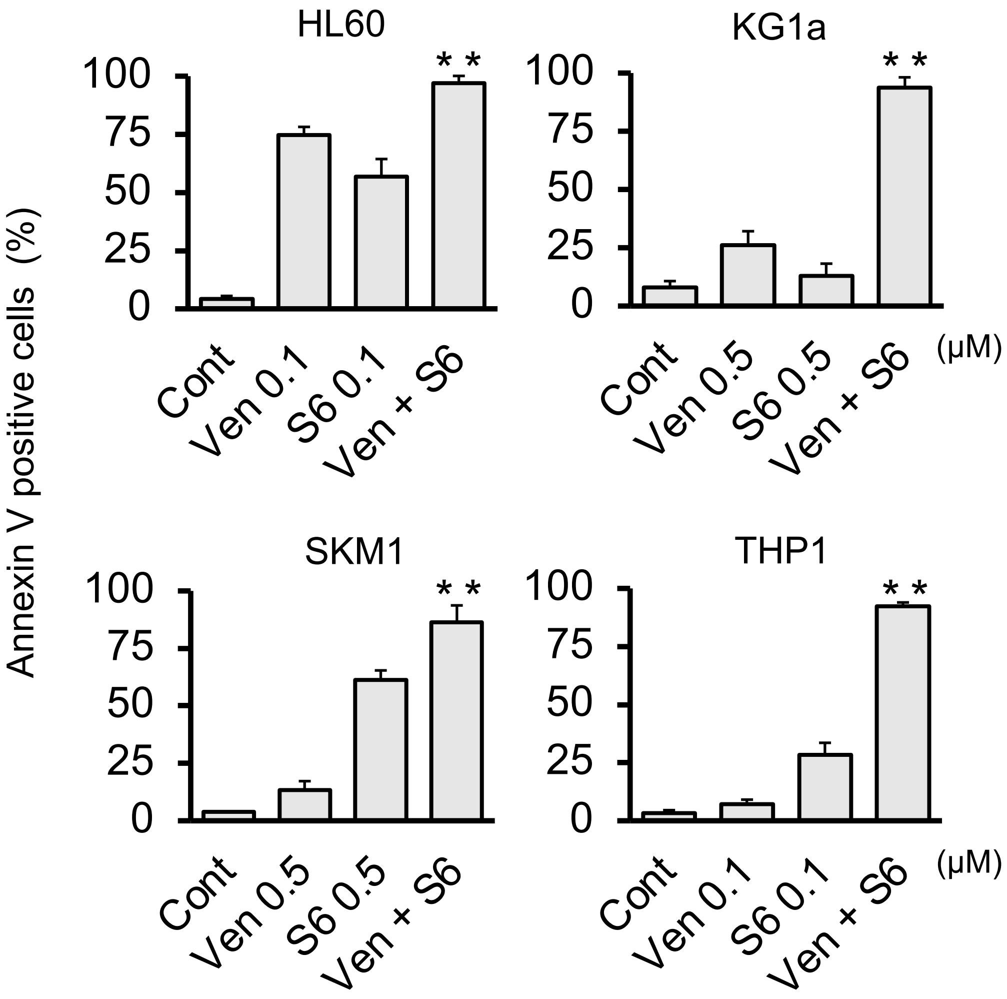

Figure S5. The combination effect of venetoclax (Ven) and S63845 (S6) in acute myeloid leukemia.

The combination of Ven plus S63845 (S6) significantly increased cell apoptosis compared with each monotherapy. Apoptosis was assessed after 72 h incubation with each drug. **$p < 0.01$.
