# Supplementary material for: Combination of a New Oral Demethylating Agent, OR2100, and Venetoclax for Treatment of Acute Myeloid Leukemia
Source: Cancer Res Commun. 2023 Feb 21;3(2):297–308. doi: 10.1158/2767-9764.CRC-22-0259 (PMC9973401; doi:10.1158/2767-9764.CRC-22-0259)
Supplement: Figure S6 — Flowcytometric analysis for ROS detection following 48 h vehicle treated (cont), 0.1 μM of azacitidine treated (A 0.1), 1.0 μM of azacitidine treated (A 1.0), 0.1 μM of venetoclax treated (V 0.1), 0.5 μM of venetoclax treated (V 0.5), 0.1 μM of azacitidine and 0.1 μM of venetoclax (A 0.1+V 0.1), 1.0 μM of azacitidine and 0.1 μM of venetoclax (A 1.0 + V 0.1), 0.1 μM of azacitidine and 0.5 μM of venetoclax (A 0.1 + V 0.5) and 1.0 μM of azacitidine and 0.5 μM of venetoclax (A 1.0 + V 0.5). [file crc-22-0259-s06.pdf]

Figure S6

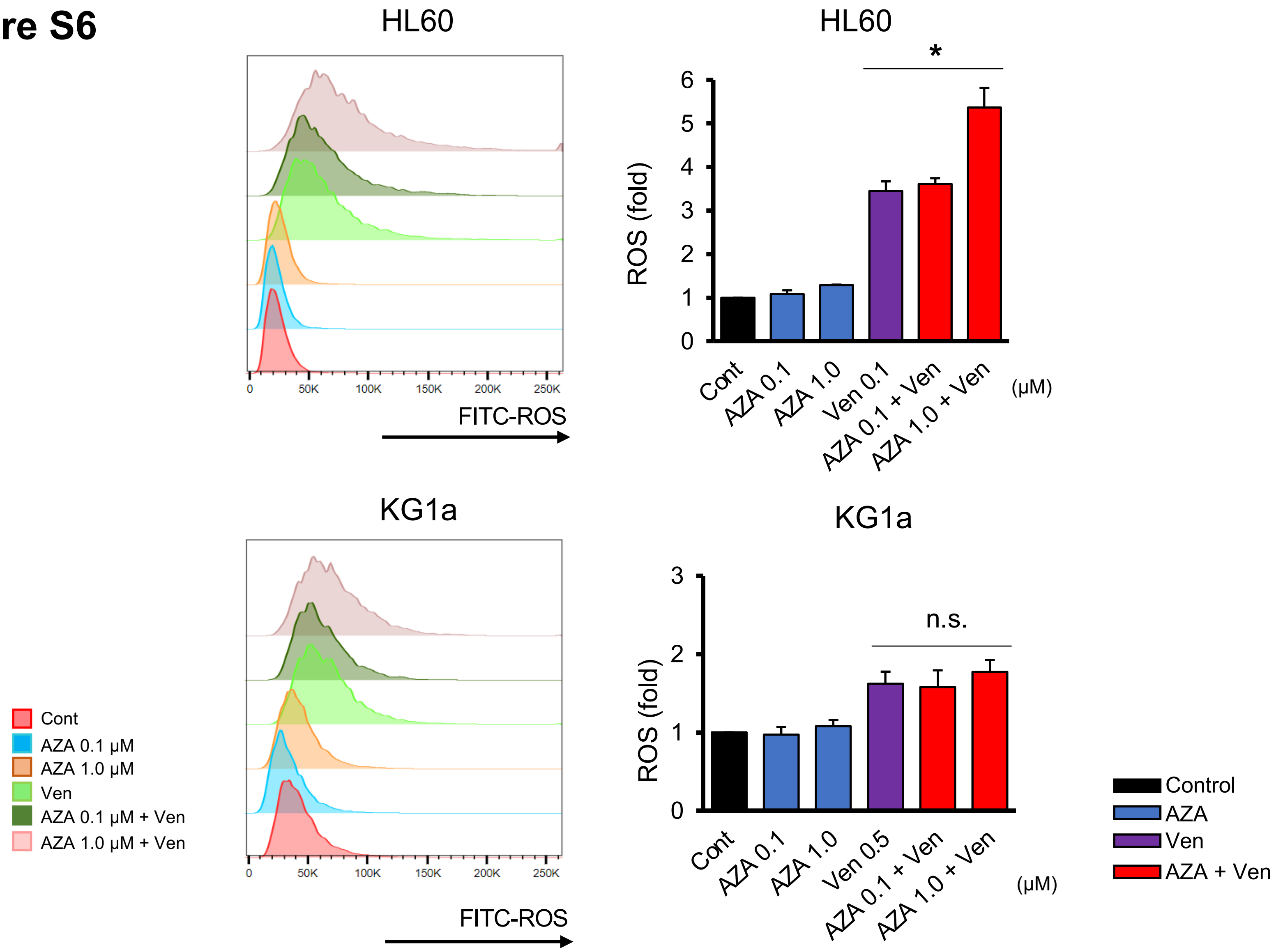

Figure S6. Flowcytometric analysis for ROS detection following 48 h vehicle treated (cont), 0.1 μM of azacitidine treated (A 0.1), 1.0 μM of azacitidine treated (A 1.0), 0.1 μM of venetoclax treated (V 0.1), 0.5 μM of venetoclax treated (V 0.5), 0.1 μM of azacitidine and 0.1 μM of venetoclax (A 0.1+V 0.1), 1.0 μM of azacitidine and 0.1 μM of venetoclax (A 1.0 + V 0.1), 0.1 μM of azacitidine and 0.5 μM of venetoclax (A 0.1 + V 0.5) and 1.0 μM of azacitidine and 0.5 μM of venetoclax (A 1.0 + V 0.5).
