# Supplementary material for: Combination of a New Oral Demethylating Agent, OR2100, and Venetoclax for Treatment of Acute Myeloid Leukemia
Source: Cancer Res Commun. 2023 Feb 21;3(2):297–308. doi: 10.1158/2767-9764.CRC-22-0259 (PMC9973401; doi:10.1158/2767-9764.CRC-22-0259)
Supplement: Figure S7 — OR21 increases ROS levels by suppressing Ven-induced mitophagy [file crc-22-0259-s07.pdf]

Figure S7

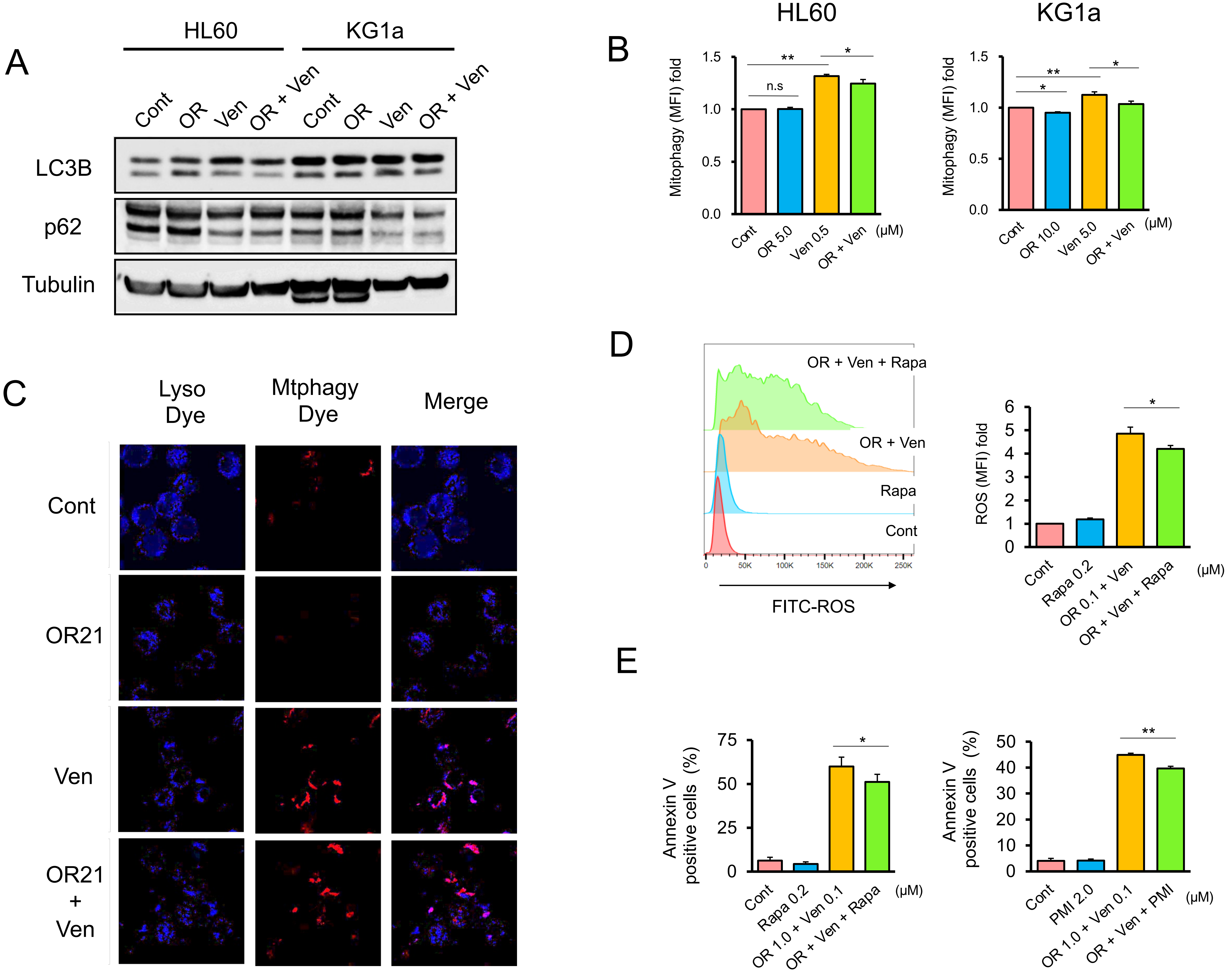

Figure S7. OR21 increases ROS levels by suppressing Ven-induced mitophagy

Immunoblots of LC3B and p62. HL60 and KG1a cells were treated 12 h with Vehicle (Cont), OR21 (1.0 μM), Ven (0.1 μM for HL60, 0.5 μM for KG1a), or OR21 + Ven (OR + Ven). Ven did not change the level of LC3B and slightly reduced p62 protein level (**A**). Flow cytometry analysis and immunofluorescence analysis show that Ven induces mitophagy; however, Ven- induced mitophagy is attenuated by addition of OR21. HL60 and KG1a cells were treated for 6 h with vehicle (Cont), OR21 (5.0 μM for HL60, or 10.0 μM for KG1a), Ven (0.5 μM for HL60, or 5.0 μM for KG1a), or OR21 + Ven (OR + Ven) (**B, C**). Additional treatment with rapamycin, a mitophagy/autophagy inducer, significantly decreased OR21 plus Ven-mediated ROS accumulation. HL60 cells were pretreated with 0.2 μM of rapamycin (Rapa) for 2 h, followed by 1.0 mM OR21 plus 0.1 μM of Ven, for 48 h (**D**). Additional pretreatment with 0.2 μM Rapa or 2.0 μM of P62-mediated mitophagy inducer (PMI) significantly decreased cell apoptosis compared with OR21 plus Ven treatment. Apoptosis was assessed after a 48 h incubation with OR21 plus Ven (**E**). *$^{*}p < 0.05$; $^{**}p < 0.01$.*
