# Supplementary material for: Combination of a New Oral Demethylating Agent, OR2100, and Venetoclax for Treatment of Acute Myeloid Leukemia
Source: Cancer Res Commun. 2023 Feb 21;3(2):297–308. doi: 10.1158/2767-9764.CRC-22-0259 (PMC9973401; doi:10.1158/2767-9764.CRC-22-0259)
Supplement: Figure S8 — Schedule of HL60 cell xenograft experiments using NOG mice. NOG mice were injected intravenously with HL60 cells. Mice were then treated with OR21 (5.4 mg/kg), Ven (25 mg/kg), and OR21 plus Ven (OR21+Ven) for 21 days since day 7 after transplantation (A). Average mouse body weights (error bars represent standard deviation) for the treatment groups were measured two times per weeks. No weight loss nor severe toxicity were observed in any group during the treatment period (B). Flow cytometric analysis showed tumor burden of human CD45+ (hCD45) leukemic cells in peripheral blood on day 28. The proportion of human CD45-positive cells in the peripheral blood of OR21 plus Ven-treated mice was not lower than that in OR21 or Ven-treated mice (C). Kaplan–Meier analysis showed OR21 plus Ven-treated mice did not exhibit prolonged survival compared to OR21 or Ven-treated mice (p = 0.308 vs. Ven; p = 1.0 vs. OR21; D). [file crc-22-0259-s08.pdf]

Figure S8

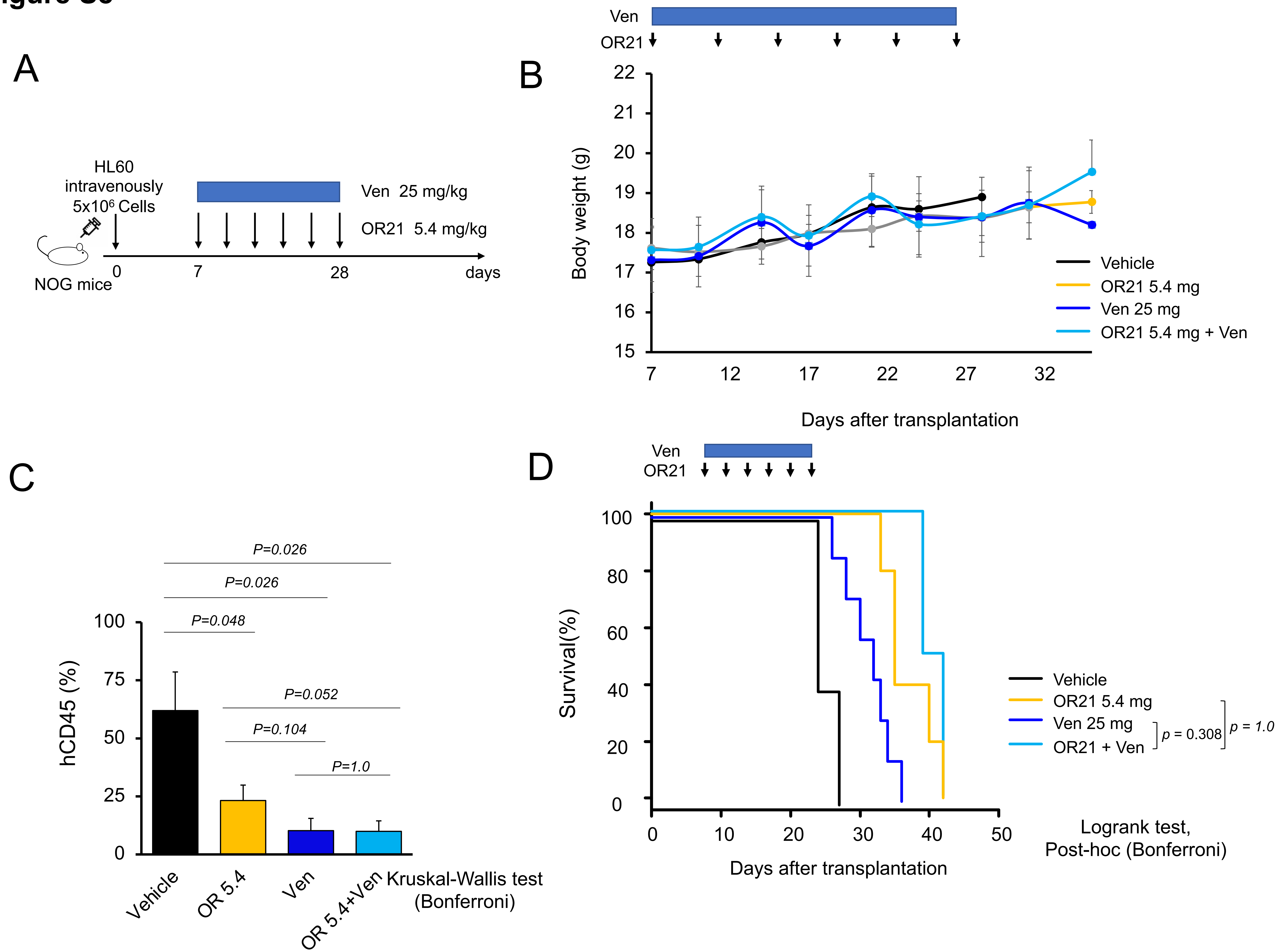

Figure S8. Schedule of HL60 cell xenograft experiments using NOG mice. NOG mice were injected intravenously with HL60 cells. Mice were then treated with OR21 (5.4 mg/kg), Ven (25 mg/kg), and OR21 plus Ven (OR21+Ven) for 21 days since day 7 after transplantation (**A**). Average mouse body weights (error bars represent standard deviation) for the treatment groups were measured two times per weeks. No weight loss nor severe toxicity were observed in any group during the treatment period (**B**). Flow cytometric analysis showed tumor burden of human CD45+ (hCD45) leukemic cells in peripheral blood on day 28. The proportion of human CD45-positive cells in the peripheral blood of OR21 plus Ven-treated mice was not lower than that in OR21 or Ven-treated mice (**C**). Kaplan–Meier analysis showed OR21 plus Ven-treated mice did not exhibit prolonged survival compared to OR21 or Ven-treated mice ($p = 0.308$ vs. Ven; $p = 1.0$ vs. OR21; **D**).
