# Supplementary material for: Combination of a New Oral Demethylating Agent, OR2100, and Venetoclax for Treatment of Acute Myeloid Leukemia
Source: Cancer Res Commun. 2023 Feb 21;3(2):297–308. doi: 10.1158/2767-9764.CRC-22-0259 (PMC9973401; doi:10.1158/2767-9764.CRC-22-0259)
Supplement: Table TS1 — IC50 in acute myeloid leukemia cell lines (µM) [file crc-22-0259-s09.pdf]

Table S1. IC_{50} in acute myeloid leukemia cell lines (μM)

Cell line	OR21	DAC	AZA	Ven
HL60	1.8	0.3	5.0	0.1
KG1a	1.4	0.5	9.3	2.5
SKM1	0.2	0.8	1.9	1.2
THP1	>10	>10	>10	0.5
Kasumi1	0.6	2.6	7.4	1.2
