# Supplementary material for: Combination of a New Oral Demethylating Agent, OR2100, and Venetoclax for Treatment of Acute Myeloid Leukemia
Source: Cancer Res Commun. 2023 Feb 21;3(2):297–308. doi: 10.1158/2767-9764.CRC-22-0259 (PMC9973401; doi:10.1158/2767-9764.CRC-22-0259)
Supplement: Table TS2 — The Bliss score in combination of venetoclax [file crc-22-0259-s10.pdf]

Table S2. The Bliss score in combination of venetoclax

Cell line	OR	DAC	AZA
HL60	24.6	10.3	26.7
KG1a	30.4	14.7	21.2
SKM1	-12	-3.7	-25.4
THP1	4.6	6.8	-12.1
Kasumi1	7.1	18.1	21.9
