# Supplementary material for: Combination of a New Oral Demethylating Agent, OR2100, and Venetoclax for Treatment of Acute Myeloid Leukemia
Source: Cancer Res Commun. 2023 Feb 21;3(2):297–308. doi: 10.1158/2767-9764.CRC-22-0259 (PMC9973401; doi:10.1158/2767-9764.CRC-22-0259)
Supplement: Table TS3 — The Bliss score in combination of S63845 [file crc-22-0259-s11.pdf]

Table S3. The Bliss score in combination of S63845

Cell line	OR	DAC	AZA
HL60	17.1	4.1	16.1
KG1a	6.8	-2.3	-1.9
SKM1	-1.4	16.6	4.4
THP1	6.7	9.2	0.7
Kasumi1	2.1	-1.1	-7.3
