# Supplementary material for: Combination of a New Oral Demethylating Agent, OR2100, and Venetoclax for Treatment of Acute Myeloid Leukemia
Source: Cancer Res Commun. 2023 Feb 21;3(2):297–308. doi: 10.1158/2767-9764.CRC-22-0259 (PMC9973401; doi:10.1158/2767-9764.CRC-22-0259)
Supplement: Table TS4 — The transcripts per million (TPM) values of six downregulated genes [file crc-22-0259-s12.pdf]

Table S4

HL60	Cont	OR	Ven	OR+Ven
RNH1	1.91	1.53	20.65	0.78
NSF	1.85	0.08	1.23	0.13
BFAR	0.26	0.53	0.6	0.87
CUTA	122.4	27.74	132.12	3.18
VAMP7	24.51	0.88	18.75	1.68
GGNBP2	2.4	0.26	3.35	0.26

KG1a	Cont	OR	Ven	OR+Ven
RNH1	0.86	6.31	4.06	1.19
NSF	0.28	0.08	0.68	0.19
BFAR	0.43	4.03	12.43	0.81
CUTA	118.88	1.89	1.66	1.6
VAMP7	7.6	10.64	16.63	5.2
GGNBP2	3.88	2.37	3.38	0.31

Table S4. The transcripts per million (TPM) values of six downregulated genes

The TPM values of six downregulated genes by OR21(OR) + venetoclax (Ven) treatment compared with Ven monotherapy are shown.
