# Supplementary material for: Combination of a New Oral Demethylating Agent, OR2100, and Venetoclax for Treatment of Acute Myeloid Leukemia
Source: Cancer Res Commun. 2023 Feb 21;3(2):297–308. doi: 10.1158/2767-9764.CRC-22-0259 (PMC9973401; doi:10.1158/2767-9764.CRC-22-0259)
Supplement: Table TS5 — The transcripts per million (TPM) values of ten upregulated genes [file crc-22-0259-s13.pdf]

Table S5

HL60	Cont	OR	Ven	OR+Ven
ASB2	0	0.04	0.09	0.8
LPCAT1	2.53	3.89	3.32	13.6
ARHGAP27	0.09	0.2	0.3	1.7
LENG8	0.29	0.07	0.07	0.4
FAM156B	0.36	0.1	0.05	6.56
PNMA5	0	2.59	0	2.05
NAP1L4	6.53	5.92	6.27	36.89
TADA2A	1.39	1.73	1.44	6.87
AL645941.3	0	0	0	37.35
AC138932.5	0.82	0.75	0.78	1.14

KG1a	Cont	OR	Ven	OR+Ven
ASB2	0.14	0.39	0.06	0.89
LPCAT1	2.9	4.13	2.99	6.78
ARHGAP27	0.25	1.98	0.15	1.8
LENG8	2.41	0.4	0.35	6.51
FAM156B	0.05	0.13	0.16	0.25
PNMA5	0	0.57	0	0.44
NAP1L4	21.62	41.38	9.08	19.88
TADA2A	1.58	2.2	2.01	4.37
AL645941.3	0.82	57.03	0	0
AC138932.5	27.64	0.35	0	33.93

Table S5. The transcripts per million (TPM) values of ten upregulated genes
The TPM values of ten upregulated genes by OR21(OR) + venetoclax (Ven) treatment compared with Ven monotherapy are shown.
